# Seismic performance analysis of a wind turbine tower subjected to earthquake and ice actions

**DOI:** 10.1371/journal.pone.0247557

**Published:** 2021-03-31

**Authors:** Shuai Huang, Yuejun Lyu, Liwei Xiu, Haijun Sha

**Affiliations:** Institute of Crustal Dynamics, China Earthquake Administration, Beijing, PR China; University of Vigo, SPAIN

## Abstract

Sea ice is one of the main loads acting on a wind turbine tower in areas prone to icing, and this threatens safe working life of the wind turbine tower. In our study, a simplified calculated model of ice, wind turbine tower, and water dynamic interaction under earthquake action was proposed, which could avoid to solve a large number of nonlinear equations. Then, the seismic behaviour of the wind turbine tower with and without the influence of sea ice was investigated, and we found that the influence of the greater mass of the sea ice on the seismic response of a wind turbine tower should be considered when the wind turbine tower is designed in an area with thick ice. With the influence of the most unfavourable ice mass, the deformation and energy dissipation capacity of the wind turbine tower are decreased, and the wall thickness or stiffening rib thickness should be increased to improve the seismic performance and ductility of the wind turbine tower; the shear force and bending moment increased significantly on the wind turbine tower, and the shear force changes at the bottom of the wind turbine tower and position of action of the sea ice: attention should be paid to the wind turbine tower design at these positions. Finally, we conducted the shaking table test, and verified the rationality of our proposed simplified model.

## 1 Introduction

Sea area in north China also coincides with seismic activity occurring during the annual four-month icing period. For example, the maximum floating ice range is from 20 to 30 nautical miles in Bohai Bay, and the maximum ice thickness reaches 600 mm. Sea ice around the pile affects the seismic response of the wind turbine tower above. How to design such a structure surrounded by ice and ensure its safe operation is a challenge faced by engineers: however, seismic load and ice load are considered separately for hydraulic structures in the *Code for design of high-rising structures* (GB50135-2019) [1], and no design method for ocean structures under combined seismic and ice loads is provided. The codes including CSA [2], Eurocode [3], API [4], *etc*. only state that it is necessary to consider the effect of ice load. CSA takes the seismic load as a rare event and ignores its superposition with the ice load. API points out that the influence of ice load on hydraulic structures in earthquake-prone areas should be paid special attention. In addition, Japanese codes [5] also point out that it is necessary to study the seismic performance of structures under ice load, however, none of these codes provide a calculation method for the coupling of the structure, ice, and water under earthquake loads.

Due to the influence of dynamic ice loading on the seismic response of structures in water in many cases [6–8], this issue has received much attention in academic and engineering circles. Paavilainen *et al*. [9] investigated the effects of ice thickness, strength, ice–ice friction, and ice plastic limit on a wide sloping structure, and found that the parameters usually interact, thus indicating that their effects cannot be considered separately. Zheng *et al*. [10] investigated the wind turbine response in dry flume, low and high calm water levels, with and without regular or random waves, and found that the peak acceleration excited by a moderate sea condition is comparable to that by a moderate earthquake. devoted to probe the joint action of strong earthquakes and moderate sea conditions. Yamauchi *et al*. [11] studied the effects of ice sheets on the seismic performance design for gravity-based structures, and found that the combination of ice load and seismic loads depends on the indentation velocity of the ice, and the interaction between the structure and ice may mitigate the seismic load. Kiyokawa *et al*. [12] presented a method for hydrodynamic pressure calculation acting on offshore structures in sea-ice region, and found that the coupling effects of fluid compressibility and ice cover have great influence on the characteristics of the hydrodynamic pressures. De Risi *et al*. [13] investigated the structural performance assessment of a typical offshore wind turbine subjected to strong ground motions, and it is observed that monopile-supported offshore wind turbines are particularly vulnerable to extreme crustal and interface earthquakes. Kouichi *et al*. [14] simplified the structure and ice system to a three-particle system and investigated the effect of structural form on the internal force response of the interactions therein. Jia [15] assessed the pier response to ice and earthquake actions through numerical analysis, and the conclusions indicate that the analytical method proposed in this paper can provide references for the development of ice mechanics, and is feasible for the study of the response of deep-water piers. Feng *et al*. [16] showed that the thick ice generated around the structure not only changes the constraints and boundary conditions of the structure, but also induces a greater force on the structure under seismic load. Ice increases the lateral restraint stiffness of the structure, thereby reducing the free length thereof. Kaynia *et al*. [17] reviewed the state of practice in seismic design of offshore wind turbines, and it is demonstrated that wind turbines are in particular vulnerable to vertical earthquake excitation due to their rather high natural frequencies in vertical direction. Qi [18] analysed the influence of consolidated ice on the seismic response of a pier under multi-point input of ground motion, and the research results show that the seismic response of the pier under the uniform excitation of ground motion is significantly greater than that without ice when the pier is surrounded by consolidated ice, and the influence of travelling wave effects cannot be ignored. Although the influence of ice load on the structure has been studied widely, few studies have been conducted into the seismic behaviour of wind turbines affected by sea ice; because such wind turbines are new structural forms, there is a lack of codified seismic design provision governing their analysis, especially for wind turbines located at sea. The effect of sea ice on the seismic behaviour of a wind turbine tower is rarely studied. Only when the seismic behaviour of the wind turbine tower affected by the ice in the sea is determined, can the safety of the wind turbine tower be ensured.

In our study, firstly, a simplified model, which could be implemented incorporating the interaction of sea ice, water, and structure, was proposed; secondly, the rationality of the simplified model and the accuracy of the calculated results were verified by shaking table test; Finally, we analysed the seismic behaviour of the wind turbine tower with and without the influence of the sea ice using our proposed simplified model.

## 2 Simplified calculation model of water-sea ice–wind turbine tower affected by earthquake

### 2.1 Establishment of the simplified calculation model

The sea ice around the engineering structure not only changes the constraints and boundary conditions of the structure, but also vibrates with the structure in the form of added mass, increasing the seismic response of the structure. The dynamic response analysis of structure in ice water involves the dynamic coupling effect of structure-ice-water. Because the analytical solution of fluid-solid coupling is still difficult to realize at present, and the analytical formula of fluid-solid coupling motion equation directly established is relatively complex, it is not convenient for practical engineering application; therefore, it is necessary to simplify the water-ice-structure interaction. Next, we simplified ice and water, respectively.

#### (1) Simplification of the ice load

**Fig 1:**
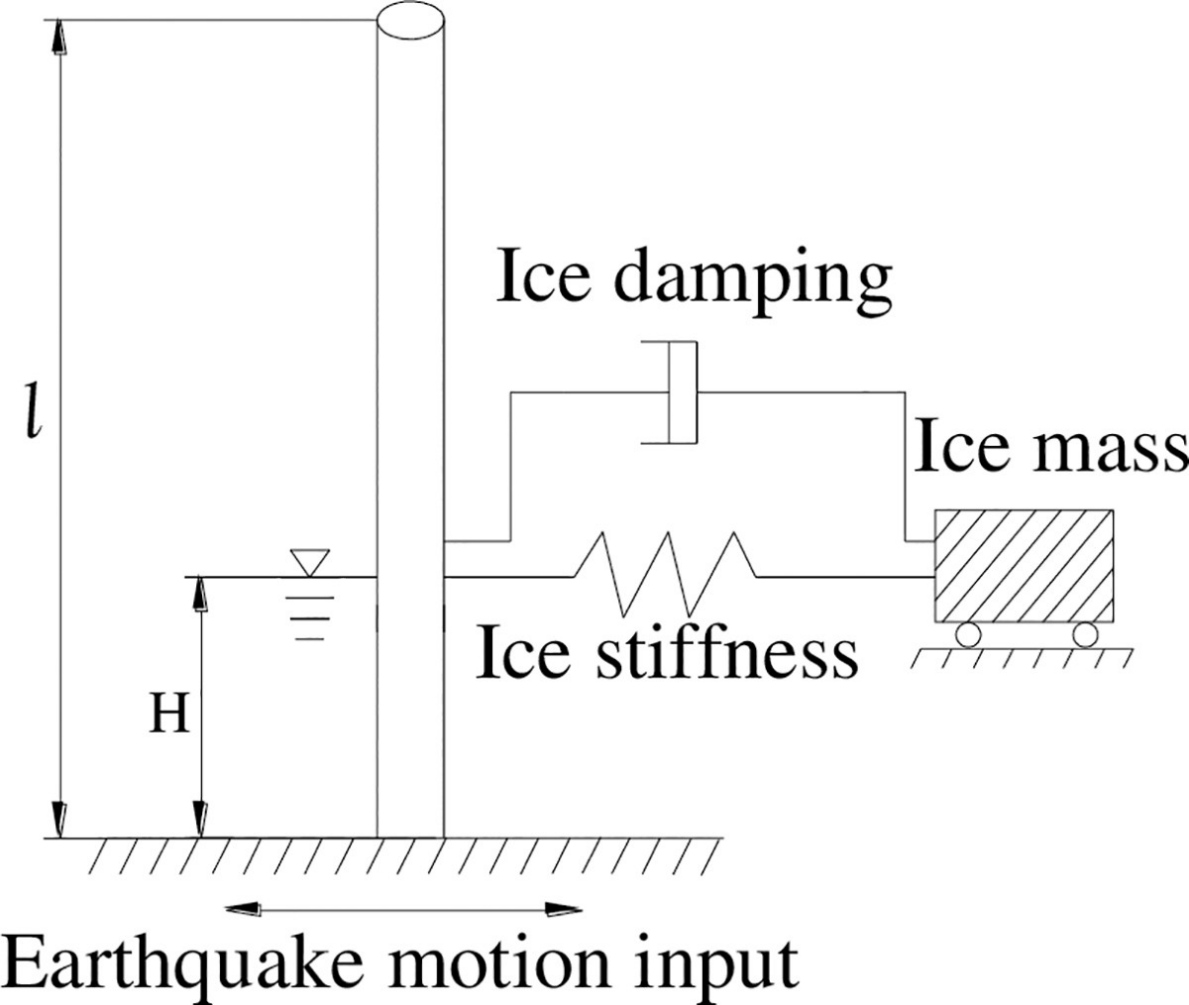
Simplified model of ice-structure dynamic interaction.

The ice, modelled in the form of a spring with certain stiffness and damping, constrains the structure at the point of consolidation, and the ice vibrates with the structure in the form of additional mass under the earthquake action. Based on the above ideas, the effect of free sea ice on the structure is considered via mass points and springs, as shown in Fig 1. Because the wind turbine is far from the coast, and the ice area is large enough, so the ice is considered as unbounded constraint, that is, the ice is unconstrained in the input direction of horizontal earthquake motion.

**Fig 2:**
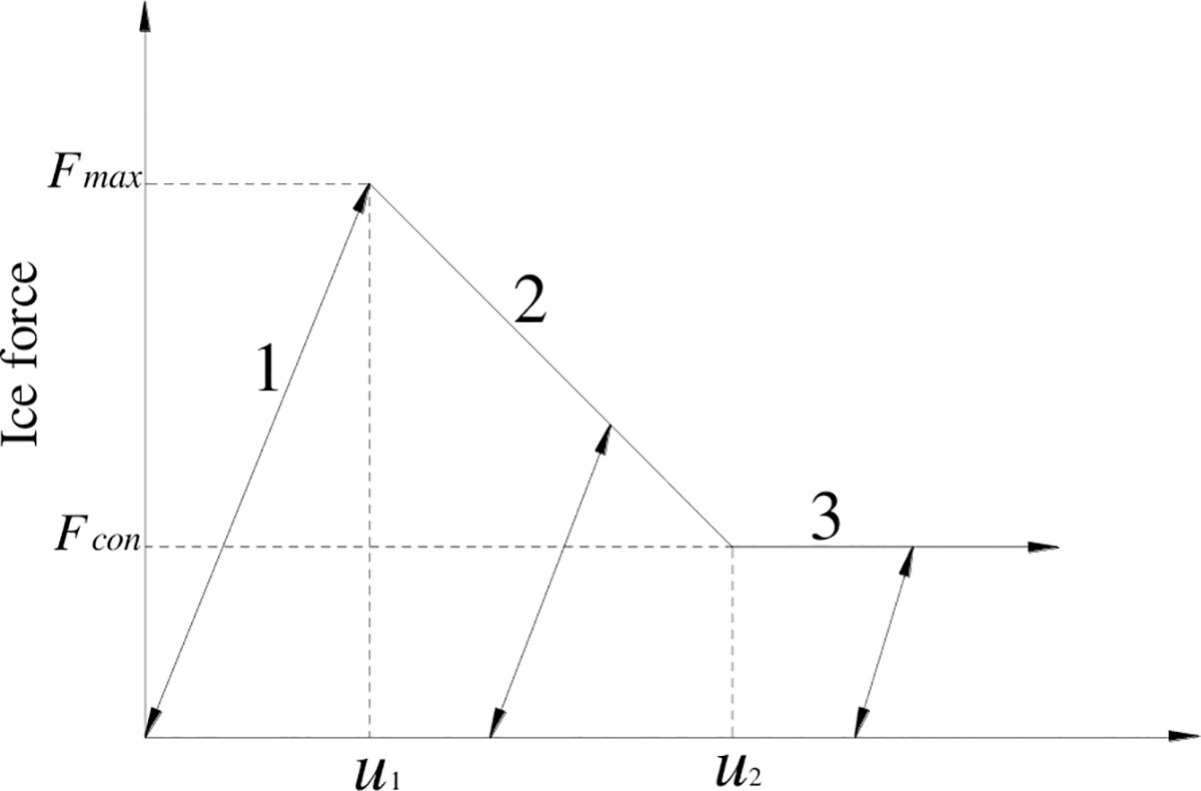
Croteau ice force model.

In our study, the added mass and spring are used to consider the sea ice load on the tower, and the spring stiffness is based on the Croteau ice force model (Fig 2). The maximum ice force in the ice force model is calculated by Korzhavin’s method [19]. The sustained ice breaking force $F_{\mathrm{con}}^{c}$ is a third of the maximum ice force $F_{\max}^{c}$. The relative displacement *u*_2_ = 2*u*_1_, and the relative displacement in the model is an important factor influencing the ice force model; however, there is no theoretical formula or empirical formula for the analysis and calculation of ice force model. The relative displacement parameters in the ice force model and the sustained breaking ice force were obtained by ice plate penetration experiment using the same parameters as numerical simulation.

#### (2) Simplification of hydrodynamic pressure

**Fig 3:**
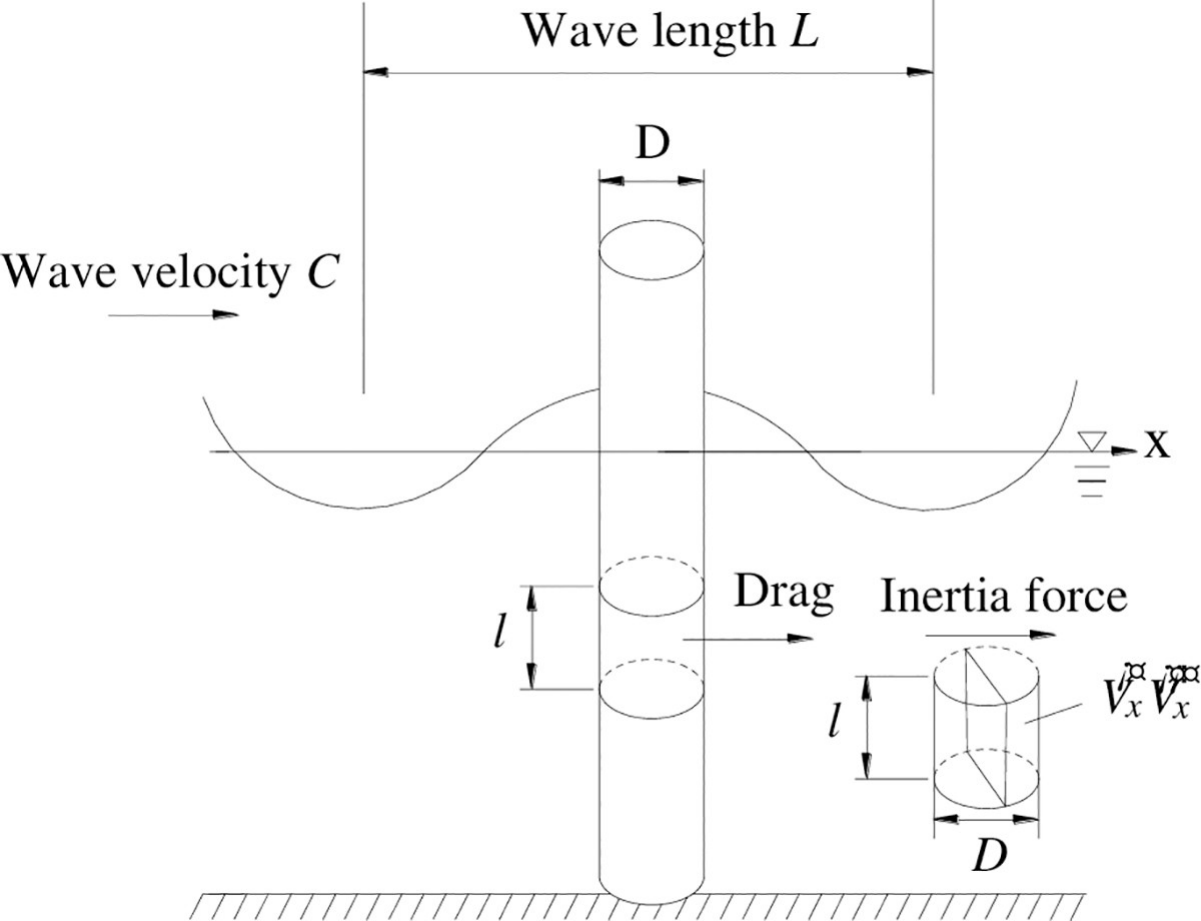
Schematic diagram of dynamic water pressure calculation.

Cylinders with a ratio of diameter *D* to wavelength *L* less than 0.2 are usually treated as small-size structures, that is, the effect of structure on fluid motion is negligible. Let the velocity of the structure in the *X* direction be $\dot{x}$ under the earthquake action, the velocity of a fluid particle is *u*, and the velocity of the fluid particle relative to the structure is $u-\dot{x}$. According to the Morison hydrodynamic theory [20], the hydrodynamic force acting on the pile consists two parts: the inertial force *F*_*I*_ produced by the acceleration or deceleration of fluid motion which is blocked by the structure when the fluid flows through the structure; Resistance *F*_*D*_ caused by the action of viscosity on the structure wall and the vortices generated by the fluid behind the cylindrical structure. The force schematic diagram is shown in Fig 3.

The inertial force is related to the relative acceleration between the fluid and the cylindrical structure that is fixed at the bottom of the water. If the deformation and vibration of the pile under the water flow action are not considered, the inertial force *F*_*I*_ is only related to the acceleration of the fluid particle. The water body occupied by the structure which is the same volume with the water body accelerates motion, and the water body will be slowed to a standstill due to the presence of the structure; therefore, there will be inertial force, which is equal to the water mass times the corresponding acceleration, acting on the cylindrical structure. Except for the part of the water occupied by the structure, there will also be additional water body near the structure, and the water body is accelerated or decelerated. The inertial force *F*_*I*_ acting on unit height of the structure at the depth *z* is shown in Eq (1) and Eq (2).

$$F_{I}=f_{I}\Delta d=(CM‐1)\rho \Delta V(\dot{u}‐\ddot{x})$$ (1)

$$f_{I}=(C_{M}-1)\rho A{\left(\dot{u}-\ddot{x}\right)}_{z}=(C_{M}-1)\rho \frac{\pi D^{2}}{4}{\left(\dot{u}-\ddot{x}\right)}_{z}$$ (2)

where *C*_*M*_ is the mass coefficient or inertia coefficient.

The resistance *F*_*D*_ is related to the relative velocity between the fluid and the structure, and it is related to the particle velocity of the fluid for a fixed rigid cylindrical structure. The resistance *F*_*D*_ is proportional to the square of the velocity of the fluid particle. The resistance *F*_*D*_ acting on unit height of the structure at the depth *z* is shown in Eq (3) and Eq (4).

$$f_{D}=C_{d}\frac{\rho D}{2}{\left(u-\dot{x}\right)}_{z}{\left|u-\dot{x}\right|}_{z}$$ (3)

$$F_{D}=f_{D}\Delta d=C_{d}\frac{\rho }{2}A_{p}(u-\dot{x})\left|\left(u-\dot{x}\right)\right|$$ (4)

where *C*_*d*_ is the resistance coefficient related to the shape of the structure and friction resistance of cylindrical structure wall; *A*_*p*_ is the vertically projected area of the cylindrical structure per unit length.

Therefore, the hydrodynamic pressure on the cylinder structure is shown in Eq (5).

$$F(x,z,t)=F_{I}+F_{D}=(C_{M}-1)\rho \Delta V(\dot{u}‐\ddot{x})+\frac{1}{2}C_{D}\rho A_{p}(u‐\dot{x})\left|u‐\dot{x}\right|$$ (5)

Because the influence of cylinder structure on water is ignored, the cylinder structure motion will not produce the movement of water, and the velocity and acceleration of water particle are both zero. The total hydrodynamic pressure on the cylinder structure per unit length in the *X* axis direction is shown in Eq (6).

$$F_{w}=-(C_{M}-1)\rho \Delta V\ddot{x}-\frac{1}{2}C_{D}\rho A_{p}\dot{x}\left|\dot{x}\right|$$ (6)

Because the resistance term at the right end of the above equation is nonlinear, it is difficult to calculate in detail, and the quasi-linearization is adopted to calculated, as shown in Eq (7).

$$\dot{x}\left|\dot{x}\right|=x_{rms}\sqrt{\frac{8}{\pi }}\dot{x}$$ (7)

where *x*_*rms*_ is the mean square of the velocity.

Then, the hydrodynamic pressure on the cylinder structure could be calculated as shown in Eq (8).

$$F_{w}=-(C_{M}-1)\rho \Delta V\ddot{x}-\frac{1}{2}C_{D}\rho A_{p}\sqrt{8/\pi }x_{rms}\dot{x}$$ (8)

#### (3) Determination of the wind load

The wind load is applied to the particle by concentrated force, and the standard value of wind load is calculated according to the current code [1], as shown in Eq (9).

$$\omega_{ki}=\beta_{zi}\mu_{s}\mu_{zBi}\omega_{0}$$ (9)

where *ω*_*ki*_ is the standard value of the wind load acting on the unit projected area at height *z* on the wind turbine tower; *ω*_0_ is the basic wind pressure, and the basic wind pressure for a 50-year return period is 0.7 kN/m^2^, according to the code [1]; *μ*_*zBi*_ represents the height variation factor of wind pressure at height *z* on the wind turbine tower; *μ*_*s*_ is the shape coefficient of the wind load; *β*_*zi*_ is the wind fluttering factor at height *z* on the wind turbine tower.

#### (4) Simplification of superstructure of the wind turbine

**Fig 4:**
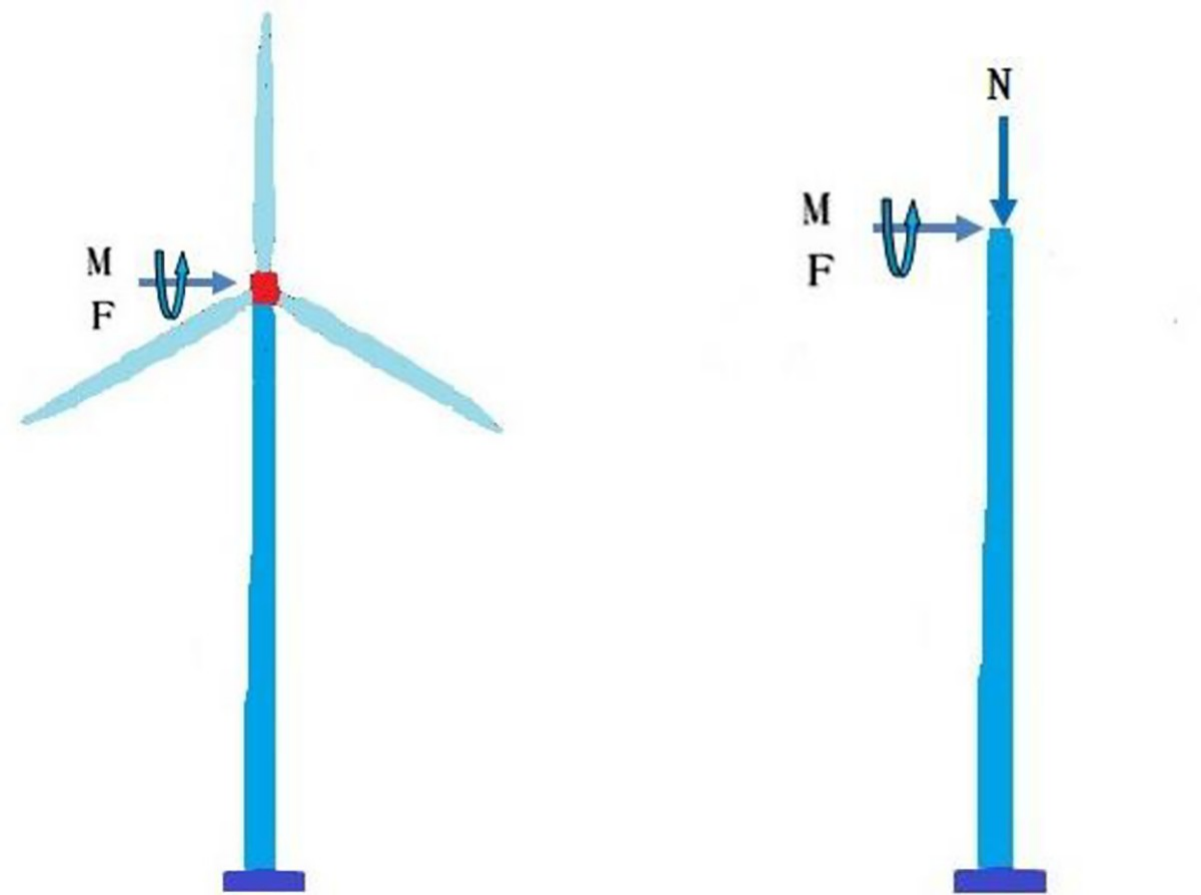
Simplification of superstructure.

In our study, the effect of the superstructure (blade, rotor, and nacelle) on the wind turbine tower is reduced to the initial bending moment, axial force and horizontal thrust, as shown in Fig 4.

The aerodynamic load of the wind turbine is calculated based on momentum theory, and the average pressure acting on the swept area of the wind rotor is calculated by Eq (10).

$$p_{H}=\frac{\rho }{2}C_{FB}V_{r}{}^{2}$$ (10)

where *C*_*FB*_ is 8/9 according to the Bates formula; *ρ* is the air density; *V*_*r*_ is the rated wind speed.

The force acting on the top of the tower is shwn in Eq (11).

$$F_{XH}=p_{H}A$$ (11)

### 2.2 Dynamic equilibrium of a monopile wind turbine tower under seismic load

**Fig 5:**
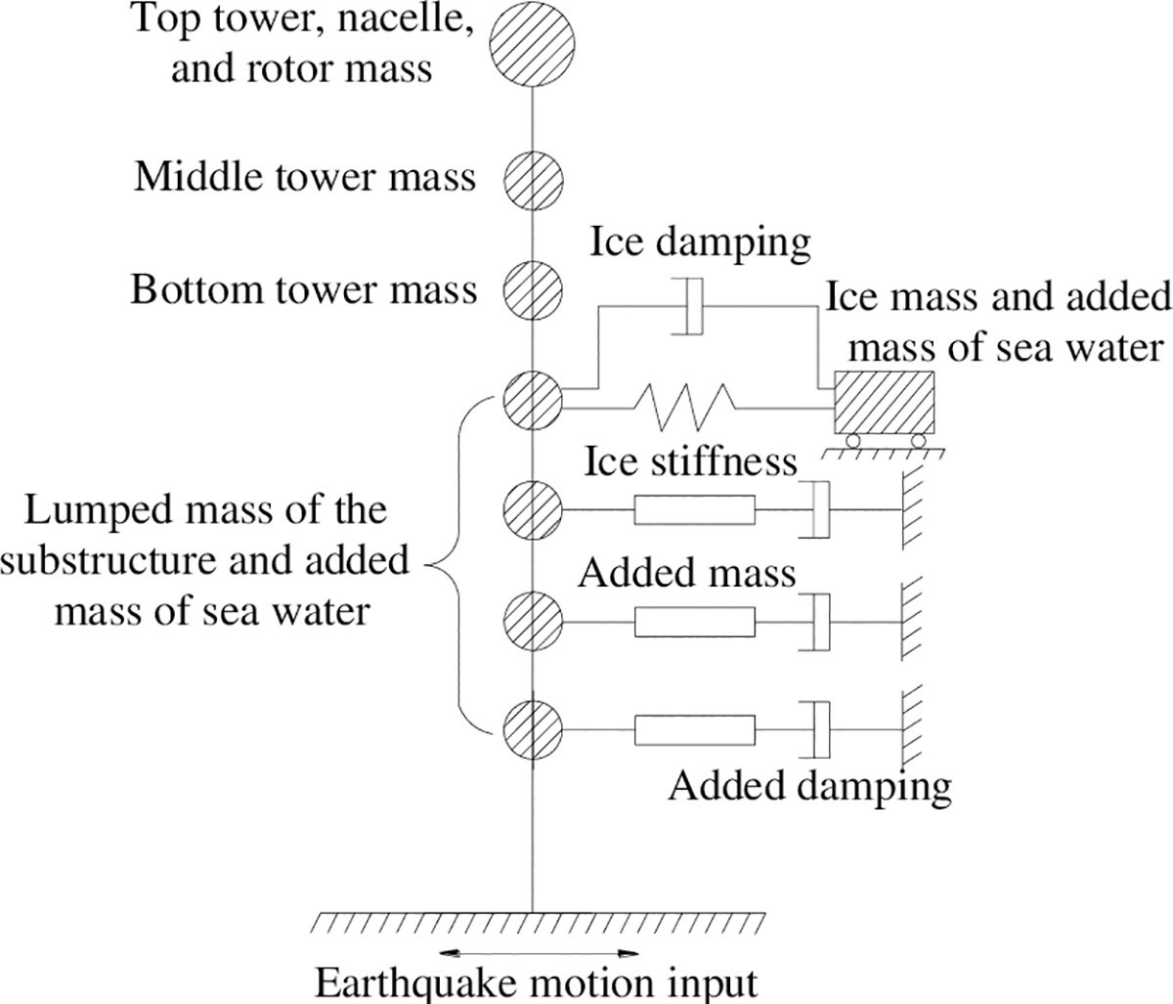
The simplified calculation model.

Based on the above ideas, the monopile wind turbine tower is simplified as a multiple-particle system, and wind turbine tower is considered as a lumped mass, and the hydrodynamic pressure is applied to the tower in the form of an added mass. The effect of sea ice on the tower is considered as a set of mass points and springs, and the input direction of earthquake motion is free and other directions are constrained. The simplified calculation model governing dynamic interaction of sea ice, water, and a wind turbine tower under seismic action is shown in Fig 5.

The time-history analysis of the wind turbine tower under earthquake action can be performed by using the acceleration input model; the sea ice and water are considered as additional masses. The calculation equation of the wind turbine tower under earthquake action is as shown in Eq (12).

$$[M]\{\ddot{x}\}+[C]\{\dot{x}\}+[K]\{x\}=-[M]\{\ddot{y}\}-[K_{Ds}]\{\dot{x}\left|\dot{x}\right|\}-W$$ (12)

where *M* is the mass matrix; *C* represents the damping matrix; *K* is the stiffness matrix; *x* is the relative displacement of the structural response; $\ddot{y}$ is the seismic acceleration; *K*_*Ds*_ is the coefficient of drag, $K_{Ds}=\frac{1}{2}C_{D}\rho A_{p}$; *W* is the wind load.

*M*_*jk*_ is the mass at the action point of the sea ice on the tower, and the dynamic equilibrium equation for *M*_*jk*_ is as shown in Eq (13):

$$\left[\begin{array}{ccccc} M_{i}+M_{ai} & 0 & 0 & 0 & 0\\ & M_{jm}+M_{ajm} & 0 & 0 & 0\\ & & M_{jk}+M_{ajk} & 0 & 0\\ & & & M_{jn}+M_{ajn} & 0\\ sym & & & & M_{u} \end{array}\right]\left\{\begin{array}{c} {\ddot{x}}_{i}\\ {\ddot{x}}_{j1}\\ {\ddot{x}}_{jk}\\ {\ddot{x}}_{jn}\\ {\ddot{x}}_{u} \end{array}\right\}+\left[\begin{array}{ccccc} C_{i} & -C_{i} & 0 & 0 & 0\\ & C_{jm}+C_{jm} & -C_{jk} & 0 & 0\\ & & C_{i}+C_{jk}+C_{k+1} & -C_{k+1} & 0\\ & & & C_{jn}+C_{u} & -C_{u}\\ sym & & & & C_{u} \end{array}\right]\left\{\begin{array}{c} {\dot{x}}_{i}\\ {\dot{x}}_{j1}\\ {\dot{x}}_{jk}\\ {\dot{x}}_{jn}\\ {\dot{x}}_{u} \end{array}\right\}$$

$$+\left[\begin{array}{ccccc} K_{i} & -K_{i} & 0 & 0 & 0\\ & K_{jm}+K_{jm} & -K_{jk} & 0 & 0\\ & & K_{i}+K_{jk}+K_{k+1} & -K_{k+1} & 0\\ & & & K_{jn}+K_{u} & -K_{u}\\ sym & & & & K_{u} \end{array}\right]\left\{\begin{array}{c} x_{i}\\ x_{jm}\\ x_{jk}\\ x_{jn}\\ x_{u} \end{array}\right\}=-\left[\begin{array}{ccccc} M_{i}+M_{ai} & 0 & 0 & 0 & 0\\ & M_{jm}+M_{ajm} & 0 & 0 & 0\\ & & M_{jk}+M_{ajk} & 0 & 0\\ & & & M_{jn}+M_{ajn} & 0\\ sym & & & & M_{u} \end{array}\right]\left\{\begin{array}{c} 1\\ 1\\ 1\\ 1\\ 1 \end{array}\right\}\ddot{y}$$

$$-\left[\begin{array}{ccccc} K_{Di} & 0 & 0 & 0 & 0\\ & K_{Dsm} & 0 & 0 & 0\\ & & K_{Dsk} & 0 & 0\\ & & & K_{Dsn} & 0\\ sym & & & & 0 \end{array}\right]\left\{\begin{array}{c} {\dot{x}}_{i}\left|{\dot{x}}_{i}\right|\\ {\dot{x}}_{jm}\left|{\dot{x}}_{jm}\right|\\ {\dot{x}}_{jk}\left|{\dot{x}}_{jk}\right|\\ {\dot{x}}_{jn}\left|{\dot{x}}_{jn}\right|\\ {\dot{x}}_{u}\left|{\dot{x}}_{u}\right| \end{array}\right\}-\left\{\begin{array}{c} 0\\ W_{jm}\\ W_{jk}\\ 0\\ W_{u} \end{array}\right\}$$ (13)

where, subscript *i* denotes the sea ice term; subscript *j* refers to the tower; *u* refers to the superstructure concentration particle; *a* refers to the added mass; *m* and *n* are positions of action of ice and tower before and after motion; *x*_*i*_ is the relative displacement of the sea ice; *x*_*j*_ is the relative displacement of the tower point-mass; *x*_*u*_ is the relative displacement of the superstructure; *W*_*u*_, *W*_*jm*_, and *W*_*jk*_ are the equivalent static wind loads corresponding to the top, middle, and bottom parts of the wind turbine tower above the sea ice action position.

For the dynamic equilibrium of the monopile wind turbine tower under seismic load, there are more mature numerical solutions available at present, including the central difference method, Wilson-*θ* method, and Newmark-*β* method. The Newmark-*β* method is an unconditional and stable implicit integral scheme. The equation could obtain the stable solution when the parameter values are 0.125 ≤ *β* ≤ 0.25 and *γ* = 0.5, and the time-step Δ*t* does not affect the stability of the solution. Accordingly, the dynamic response of the monopile wind turbine tower under earthquake action could be obtained when the time history of the earthquake load is known.

## 3 Verification of our proposed model by shaking table test

To verify the accuracy of the simplified calculation model of water-sea ice–wind turbine tower affected by earthquake based on the added mass method, shaking table tests of the wind turbine tower were undertaken. The model installed on the shaking table can actually experience the earthquake process, and the shaking table test can reproduce the entire process of wind turbine tower failure under the earthquake action [21, 22]. At the same time, calculation results of our proposed simplified model are compared with experimental data to verify the feasibility of our proposed model.

### 3.1 Test model

**Fig 6:**
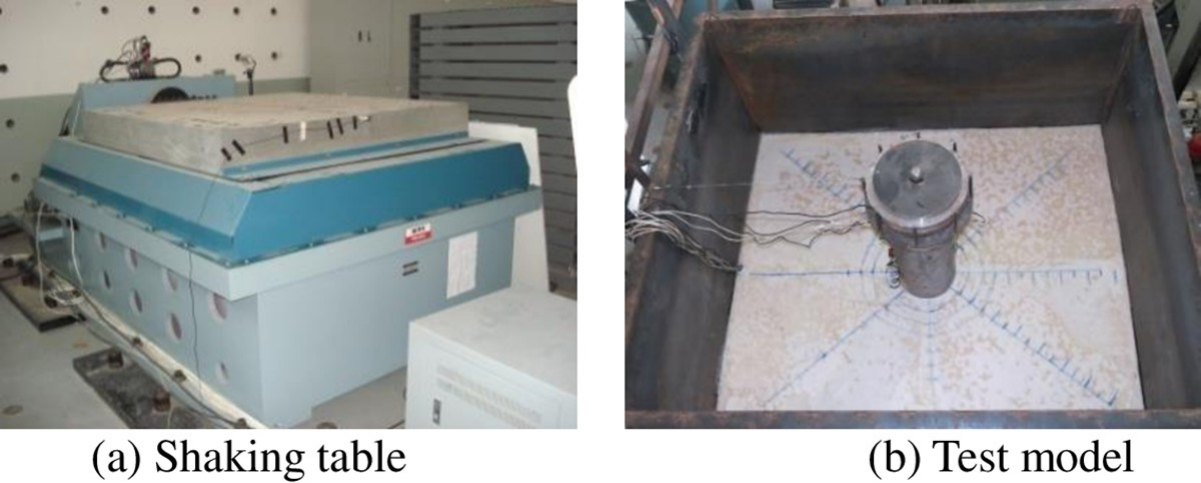
Shaking table simulation test system (ES-15).

The experiment was conducted on a shaking table simulation test system (ES-15) including a: shaking table controller, shaking table, and hydraulic pump. The main technical parameters of the shaking table test equipment include its rated working frequency (100 Hz), maximum acceleration (20 m/s^2^), maximum payload (5000 kg), and the dimensions of the shaking table (1.5 m×1.5 m). This experiment was designed to simulate the seismic dynamic interaction between the ice and structure to verify the accuracy of the simplified calculation model of water-sea ice–wind turbine tower affected by earthquake based on the added mass method, so a large water tank was fabricated and fixed onto the shaking table to hold water and ice. The dimensions of the water tank are 2.0 m (length)×1.3 m (width)×1.5 m (height). The height of the prototype structure is 80 m, and the upper structure mass is 265,585 kg. Subject to the size of the test equipment, test water boundary conditions are limited, and the similarity coefficient is *λ*_*l*_ = 1:50 to simulate the infinite boundary conditions pertaining to the water. The model height is 1.6 m, and the base of the model is welded to steel plate of the tank, as shown in Fig 6.

According to the code, the wind turbine is allowed some structural components to respond plastically under the rare earthquakes, but the structure can’t collapse. At this time, the wind turbine will stop automatically. the similarity law of elastic force-gravity is generally adopted, which ensures the consistency of the Cauchy constant and Froude constant between the test model and the prototype structure.

$$\frac{\lambda_{E}}{\lambda_{g}\cdot \lambda_{\rho }}=\lambda_{l}$$ (14)

Where *λ*_*E*_, *λ*_*g*_, *λ*_*ρ*_, *λ*_*l*_ are elasticity modulus similarity ratio, gravity acceleration similarity ratio, density similarity ratio, and geometric similarity ratio. *λ*_*l*_ = *l*_*m*_/*l*_*p*_, *m*, *p* represents the model and the prototype, respectively.

The shaking table test is carried out under the original gravity acceleration, and the gravity acceleration of the model and the prototype structure is equal. According to Buckingham π, the similarity relation of the main physical quantities of the model is derived by the dimensional method, as shown in Table 1.

**Table 1:** Similarity relation of the main physical quantities.

Physical quantity	Similarity coefficient	Physical quantity	Similarity coefficient
Length	*λ*_*l*_	Strain	*λ*_*ε*_ = *λ*_*σ*_/*λ*_*E*_
Density	*λ*_*ρ*_	Inertia force	*λ*_*F*_ = $\lambda_{\rho }\lambda_{l}^{3}\lambda_{a}$
Acceeration	*λ*_*a*_	Pressure	*λ*_*P*_ = *λ*_*E*_
Poisson’s ratio	*μ*	Bending moment	*λ*_*M*_ = $\lambda_{E}\lambda_{l}^{3}$
Time	$\lambda_{t}=\lambda_{l}\sqrt{\lambda_{\rho }/\lambda_{E}}$	Frequency	*λ*_*f*_ = 1/*λ*_*t*_
Mass	*λ*_*m*_ = $\lambda_{\rho }\lambda_{l}^{3}$	Dispacement	*λ*_*u*_ = *λ*_*l*_
Elasticity modulus	*λ*_*E*_	Velocity	*λ*_*V*_ = *λ*_*l*_/*λ*_*t*_
Stress	*λ*_*σ*_ = *λ*_*E*_		

The similar design of the model structure follows the following basic principles:

1. Ice, water, and structure follow the same similarity relationship.
2. Ensure that the gravity of the structure is not distorted, and the mass of the upper structure is also added to the top of the model in the form of mass blocks on the basis of no influence on the stiffness of the structure.
3. Control the applied dynamic load parameters to meet the performance requirements of the loading equipment.

According to the design principle of the similarity ratio, non-prototype materials were used in the test, and the elastic force-gravity similarity law was followed. The scaling law between our test model and the actual projects follows the Buckingham-π theorem [23]. The experimental design model is in the elastic response stage, so the model material should be consistent with the basic assumption of general elastic theory as far as possible. Therefore, similar models can be designed with steel materials. The similarity relations of the main physical quantities of the test model were derived by dimensional analysis (Table 2).

**Table 2:** Similarity coefficient of the test model.

Item	Physical quantity	Similarity coefficient
Geometric relationship	Length	*λ*_*l*_ = 0.02
	Displacement	*λ*_*u*_ = *λ*_*l*_ = 0.02
	Area	$\lambda_{A}=\lambda_{l}^{2}$ = 4×10^−4^
Material relationship	Elastic modulus	*λ*_*E*_ = 6.2
	Poisson’s ratio	*μ* = 1.5
	Stress	*λ*_*σ*_ = *λ*_*E*_ = 6.2
	Strain	*λ*_*ε*_ = *λ*_*σ*_/*λ*_*E*_ = 1
	Density	*λ*_*ρ*_ = 3.12
Dynamic relationship	Time	$\lambda_{t}=\lambda_{l}\sqrt{\lambda_{\rho }/\lambda_{E}}$ = 0.0142
	Frequency	*λ*_*f*_ = 1/*λ*_*t*_ = 23.81
	Velocity	*λ*_*V*_ = *λ*_*l*_/*λ*_*t*_ = 1.41
	Acceleration	1
Load relationship	Point load	*λ*_*M*_ = $\lambda_{E}\lambda_{l}^{2}$ = 2.48×10^−3^
	Bending moment	*λ*_*M*_ = $\lambda_{E}\lambda_{l}^{3}$ = 1.98×10^−4^
	Inertia force	*λ*_*F*_ = $\lambda_{\rho }\lambda_{l}^{3}\lambda_{a}$ = 1.99×10^−4^

### 3.2 Ice material selection

According to the theory that ice and structure interaction mainly occurs compression failure, the physical parameter of ice which have a major influence on the test is mainly the compression strength of the ice. Compressive strength of fully refined paraffin wax is 1.33Mpa, which is basically consistent with the extreme values of the compressive strength of the ice in the south yellow sea, which are 1.31~2.18Mpa. Therefore, the fully refined paraffin wax was used to replace natural ice.

**Fig 7:**
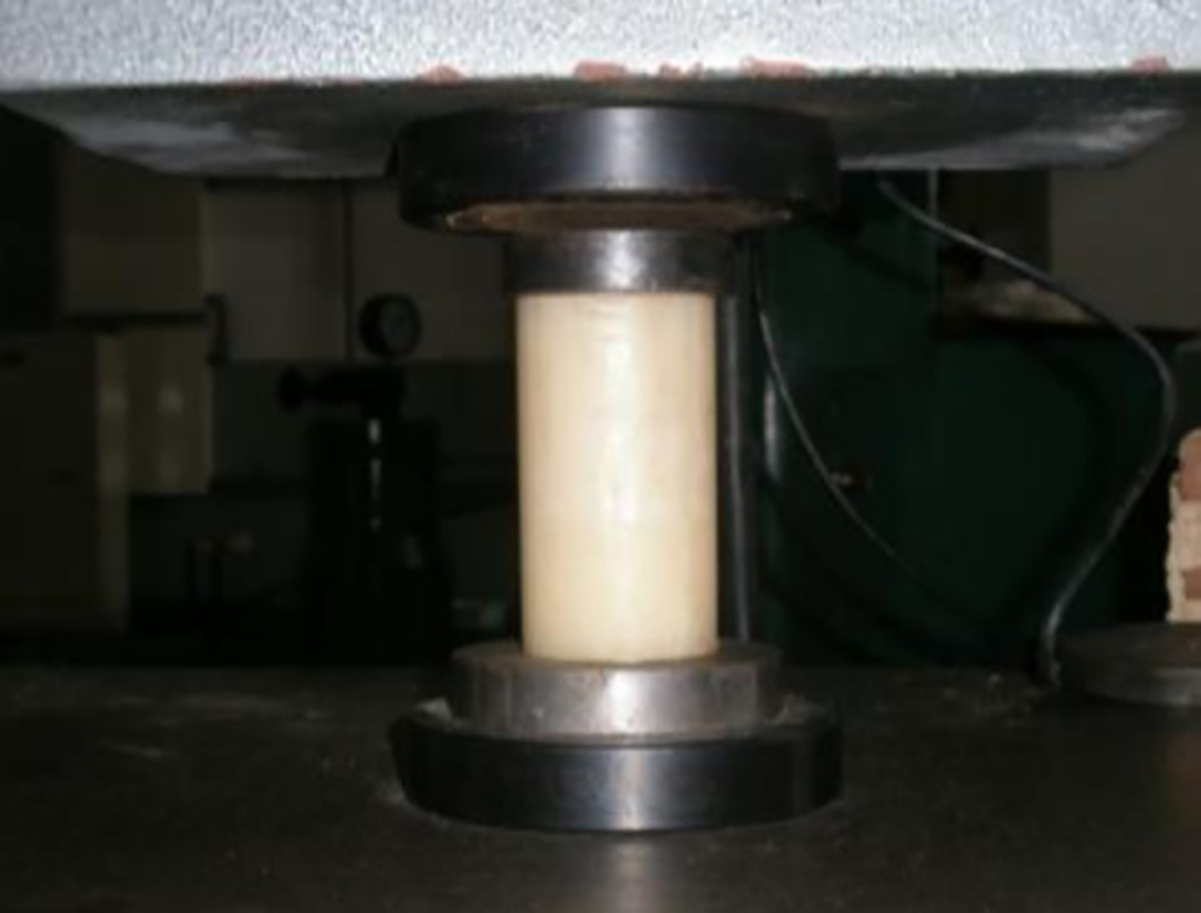
Ice samples.

There are different types of paraffin, and it can be divided into full-refined paraffin and semi-refined paraffin and coarse paraffin according to the different processing of refined degree. In order to determine the Paraffin which is similar to the stress-strain behavior of the ice, we chose four samples (1#, 2#, 3#, and 4#). The compressive strength of the paraffin wax was measured by use of the small-specimen method, as shown in Eq (15):

$$\sigma_{cu}=\frac{P_{f}}{\pi r^{2}}$$ (15)

where *σ*_*cu*_ is the uniaxial compressive strength of the sample; *P*_*f*_ is the compressive load applied at failure; *r* is the radius of the sample. Paraffin ice is cut into small cylindrical blocks, and the paraffin ice is compressed to destruction, as shown in Fig 7.

The compression of the sample was recorded at 0.2 kN increments, and the compressive strength of each sample was calculated according to the crushing load (Table 3).

**Table 3:** Compressive strength of each sample.

No.	1	2	3	4
Compressive strength (kPa)	1076.52	1112.35	1268.76	1230.34

**Fig 8:**
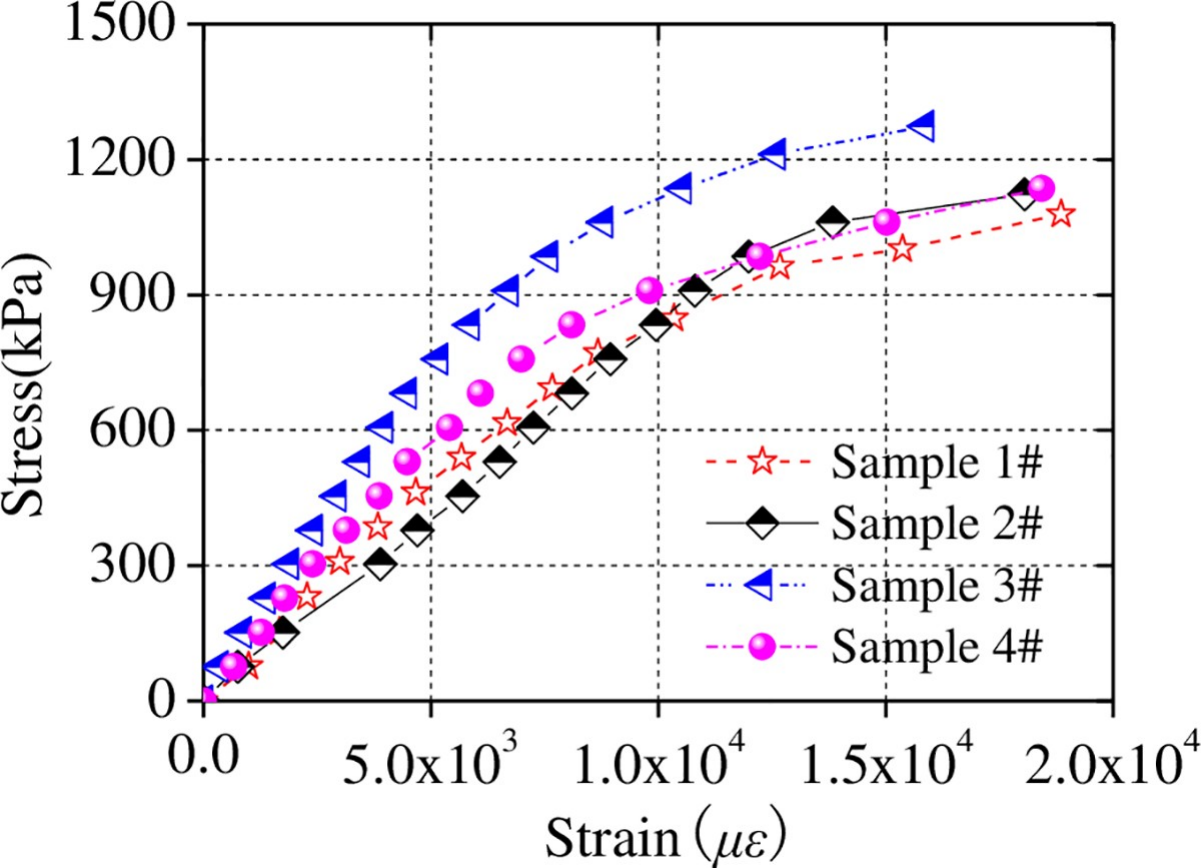
Stress-strain data.

The pressure and corresponding displacement were recorded every 0.2 kN and converted into stress and strain, as shown in Fig 8.

In the initial stage of the compression test, the ice sample is in its elastic working stage, and the elastic modulus is as listed in Table 4.

**Table 4:** Elastic modulus of each sample.

No.	1	2	3	4
Elastic modulus (MPa)	100.54	84.66	156.94	117.65

As shown in Table 4, the uniaxial compression strength of Sample 4 is approximately equivalent to that of ice, so it was used to simulate ice in the present study. The thickness of the ice was set to 24 mm according to the aforementioned similarity relationship.

### 3.3 Experimental scheme

**Fig 9:**
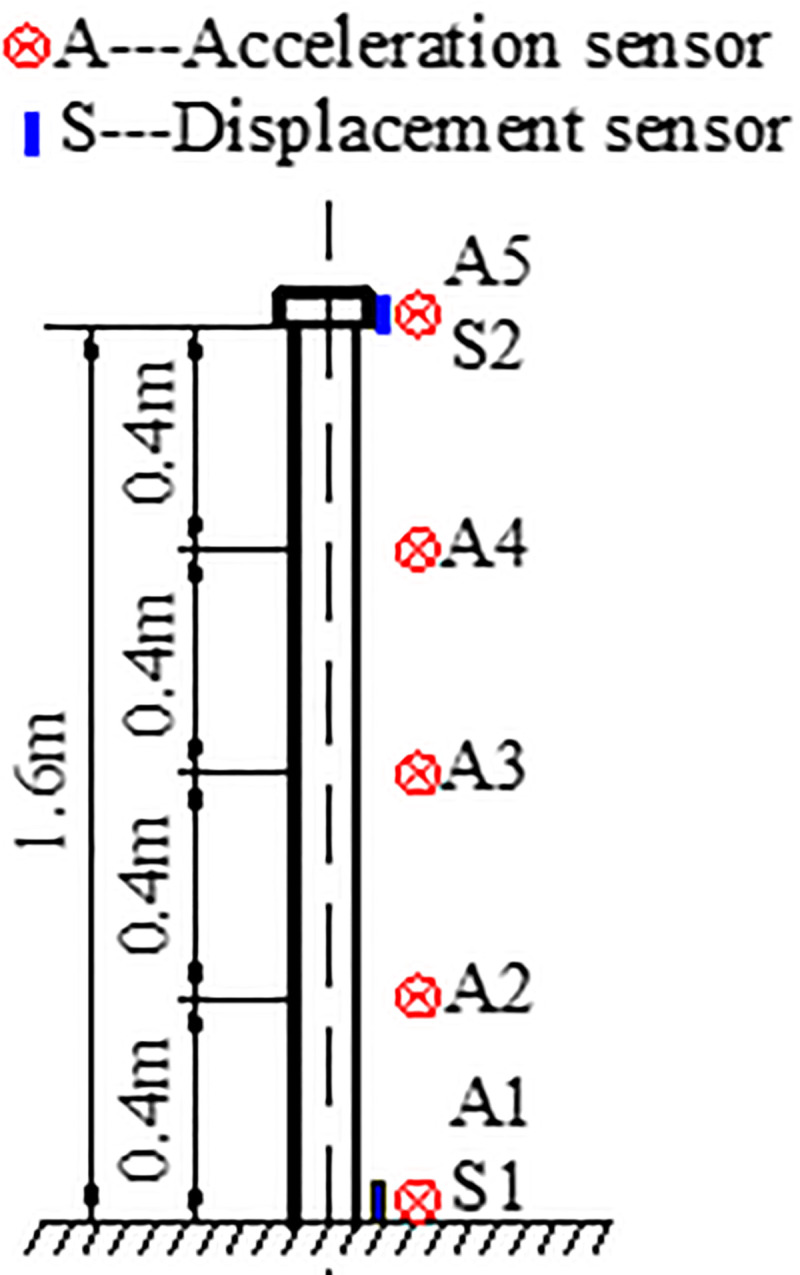
Diagram of the sensor assignments.

To measure the dynamic response of the model, four acceleration sensors were placed along the model height, and two displacement sensors were placed at the top and bottom of the model (Table 5). The diagram of the sensor assignments is shown in Fig 9.

**Table 5:** Sensor assignments.

Name	Model	Number	Description
Acceleration sensor	LC0701-5M	A1, A2, A3, A4, A5	Record the acceleration of the model at different heights
Displacement sensor	SW-1	S1, S2	Record the displacement of the model bottom and top (Range ± 100 mm)

The Tianjin seismic wave and Kobe seismic wave were selected as the input load to simulate seismic action: the Tianjin wave is an artificial earthquake, and the Kobe seismic wave is an actual seismic record. At the same time, sinusoidal waves were used to study the effect of different frequencies of vibration on the model, and the test conditions with, and without, the influences of ice are summarised in Table 6.

**Table 6:** Test conditions.

Test conditions	Seismic wave type	Peak acceleration (*g*)
SIN-01	1-Hz sine wave	0.10
SIN-02	3-Hz sine wave	0.10
Kobe-01	Kobe earthquake	0.10
Kobe-02	Kobe earthquake	0.20
TJ-01	Tianjin earthquake	0.10
TJ-02	Tianjin earthquake	0.20

The Tianjin seismic wave was recorded during the Tangshan earthquake in 1976 in China, and the Kobe seismic wave was recorded in the Kobe earthquake in 1995 in Japan. The parameters of the two seismic waves are listed in Table 7.

**Table 7:** Parameters of the two seismic waves.

Name	Earthquake	Magnitude	Epicentral distance (km)	Record location	Peak acceleration (m/s^2^)
Tianjin	Tangshan earthquake (1976)	7.5	65	Tianjin hospital	1.4580
Kobe	Kobe earthquake (1995)	7.2	11	Takatori station	6.86831

### 3.4 Analysis of test results

**Fig 10:**
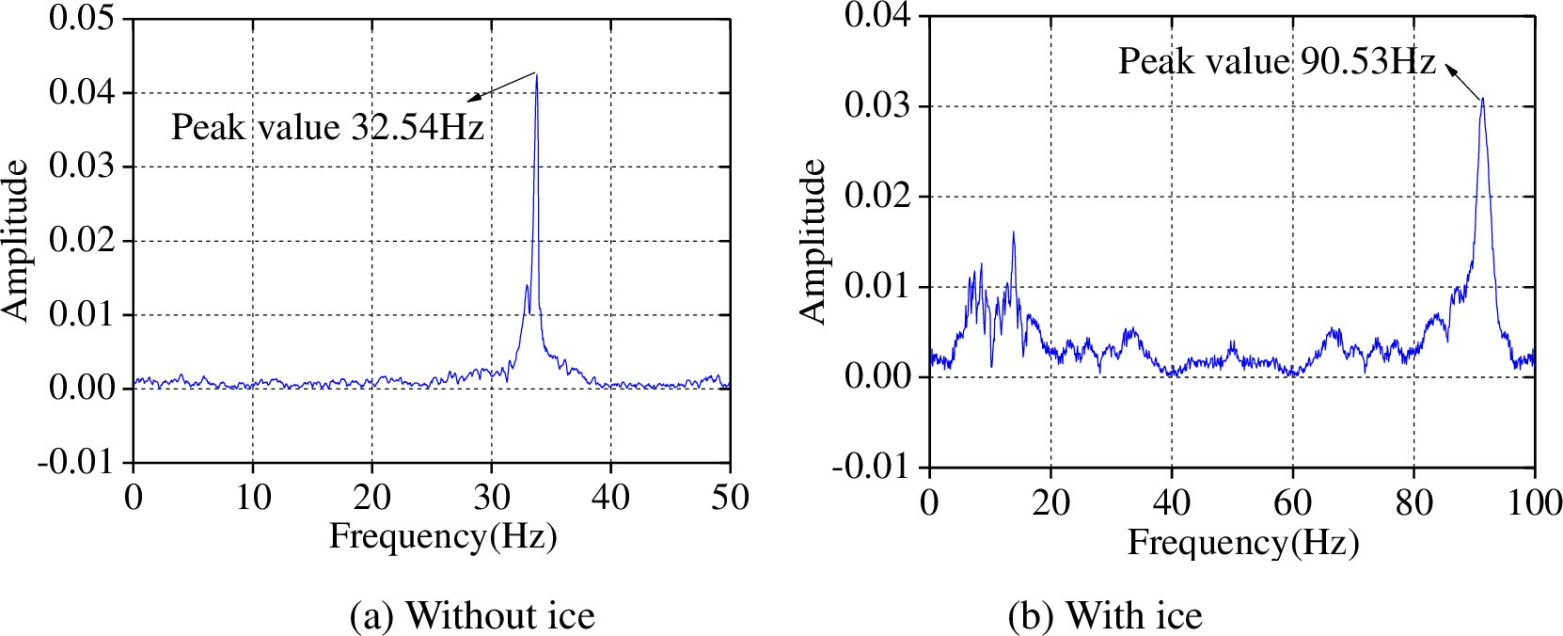
Natural vibration frequency obtained through the frequency sweep test.

In the experiment, the dynamic characteristics of the model with, and without, the influence of ice was measured by hammer decay model test and sine-wave sweep test, respectively: first order vibration frequencies of the models were obtained (Fig 10).

As shown in Fig 10, the natural vibration frequency of the test model with ice is higher than that without. The natural vibration frequency of the test model with ice is 2.78 times greater than that without: this is mainly due to the restraint imposed by the sea ice which increases the lateral restraint stiffness of the model.

**Fig 11:**
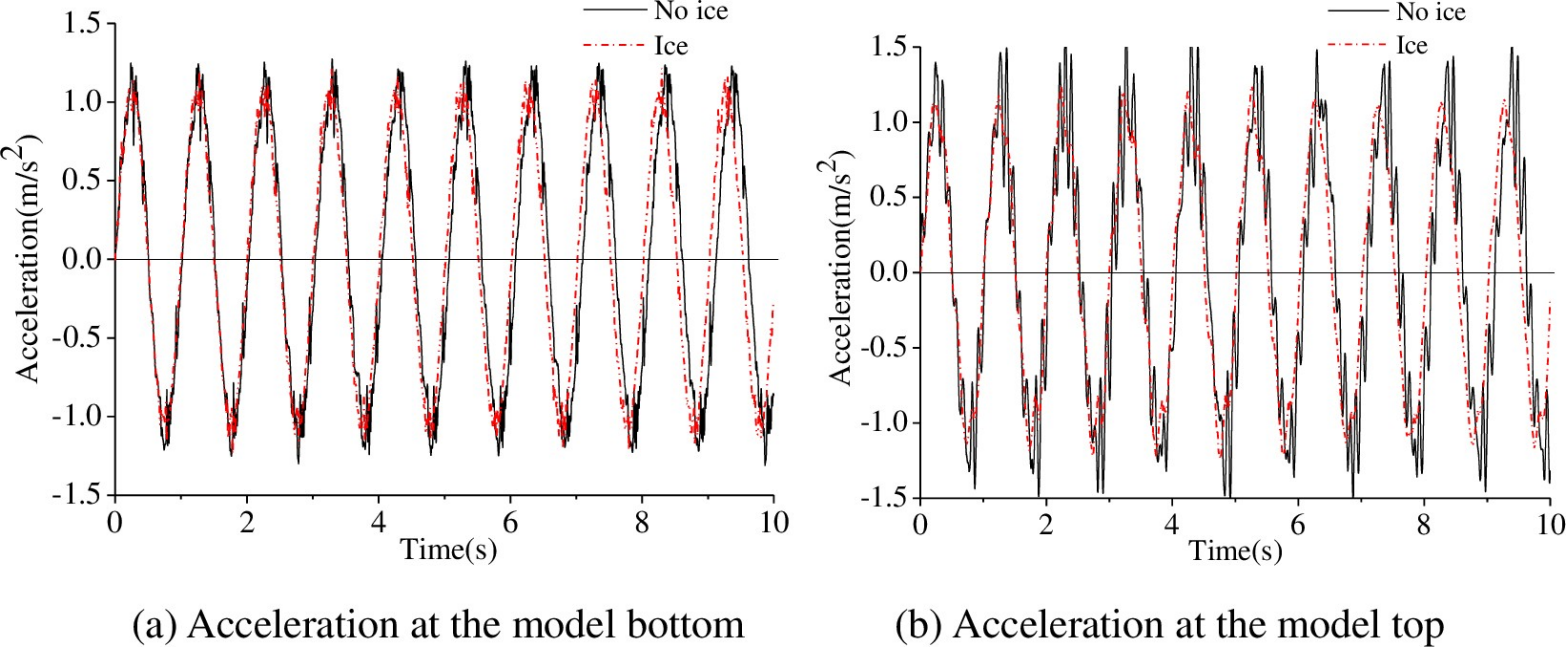
Acceleration under sinusoidal input at 1 Hz.

**Fig 12:**
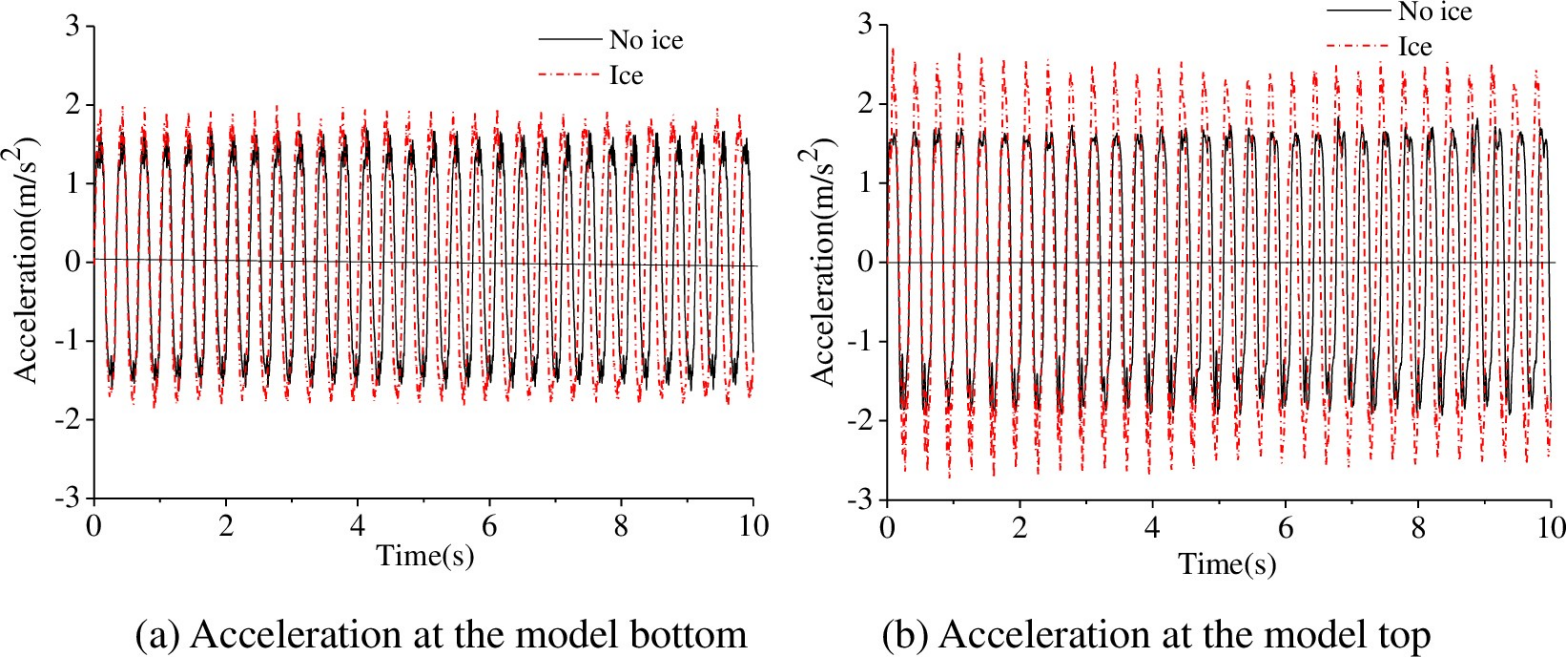
Acceleration under sinusoidal input at 3 Hz.

**Fig 13:**
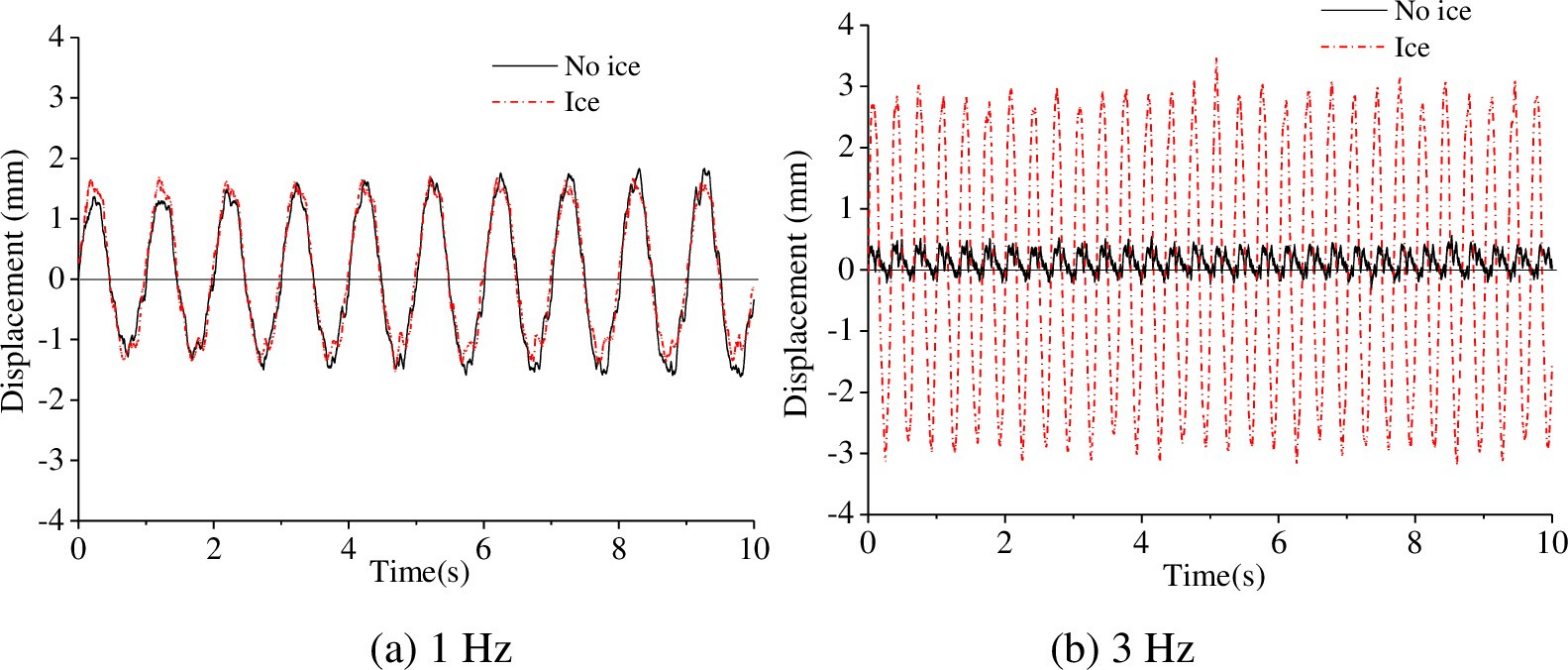
Displacement under sinusoidal input at 1 Hz and 3 Hz.

The acceleration and displacement of the model were calculated when the sinusoidal input frequencies were 1 Hz and 3 Hz are shown in Figs 11–13.

As shown in Figs 11 to 13, the acceleration response at the model bottom and top is similar, and the model underwent quasi-rigid body motion under sinusoidal excitation at 1 Hz. The relative displacement at the model bottom and top is similar; therefore, the model motion was deemed rigid under sinusoidal input excitation at 1 Hz, and the ice had little effect on the dynamic response of the model. The acceleration and the displacement under the influence of the ice are greater than that without under sinusoidal excitation at 3 Hz, which shows that the model undergoes elastic vibration. The sinusoidal vibration test results indicate that the ice load has a significant effect on the structure. In addition, the acceleration and the displacement at the model top are much greater than that at the model bottom, which shows that the vibration of the model is amplified over the model height.

To verify the accuracy of our proposed simplified calculation model, we compared the test results and the calculated results using the proposed simplified calculation model. First, we compared the natural frequencies (Table 8).

**Table 8:** Frequency comparison between test and calculated results.

Method	No ice		Ice	
	Frequency (Hz)	Period (s)	Frequency (Hz)	Period (s)
Test result	32.54	0.030	90.53	0.011
Calculated result	31.12	0.032	96.87	0.010
*R* (%)	-4.36	6.67	7.00	-9.09

**Fig 14:**
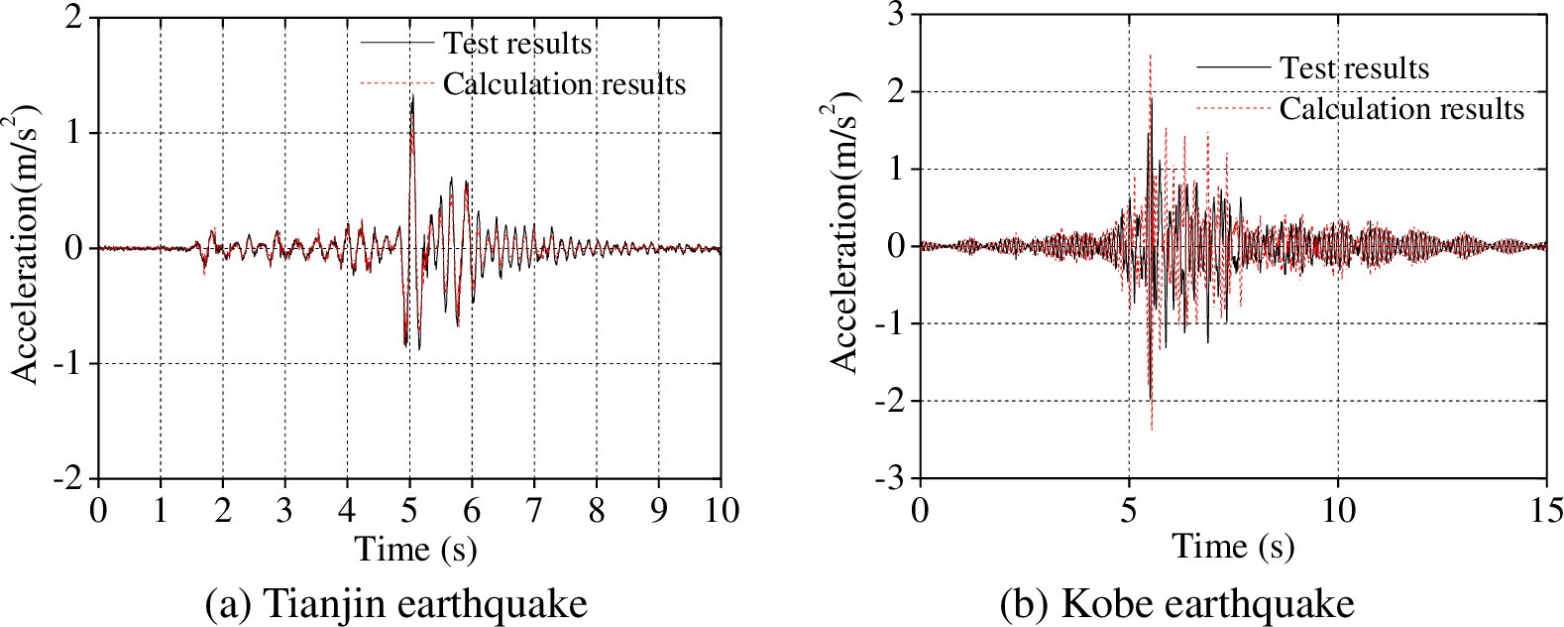
Acceleration of the model top under earthquake load (0.1*g*).

**Fig 15:**
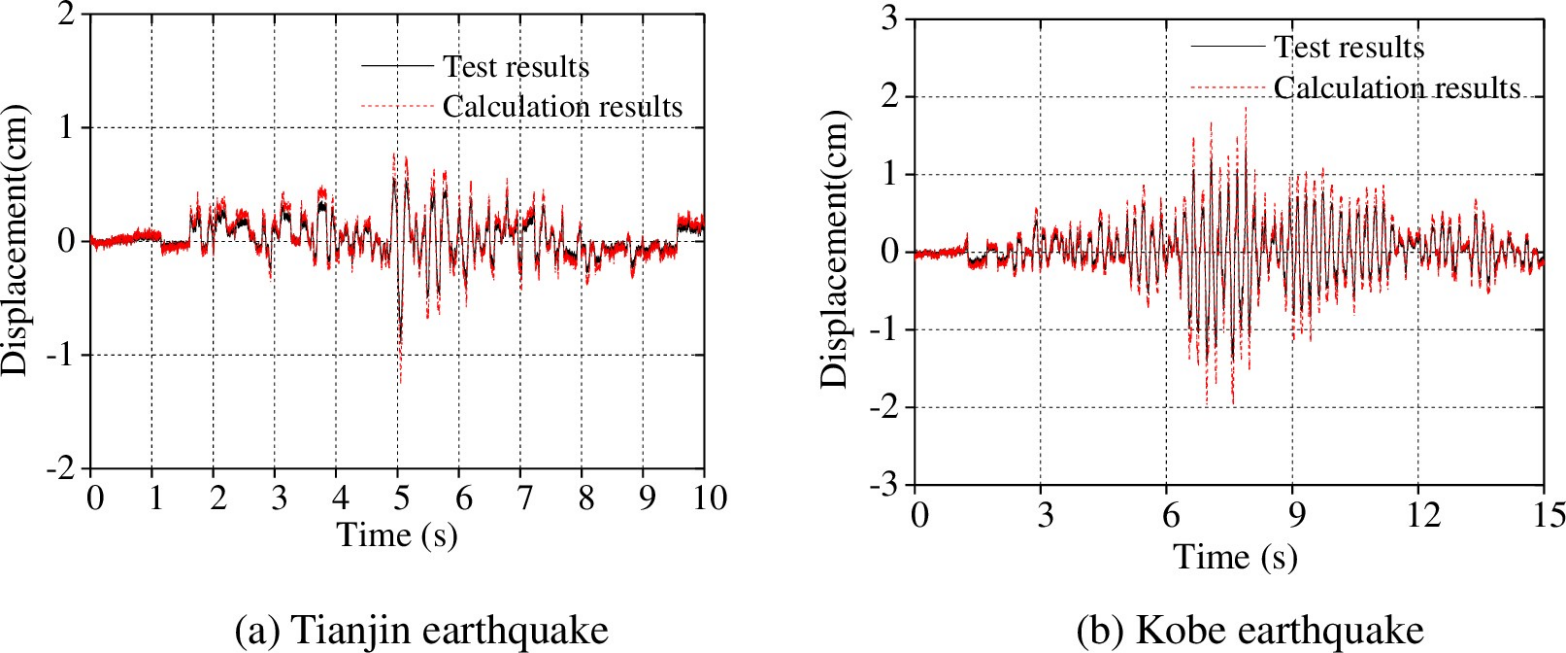
Displacement of the model top under earthquake load (0.1*g*).

As shown in Table 8, the discrepancy between measured and calculated frequencies using our proposed simplified calculation model is within ± 7% when ignoring the influence of the ice: the discrepancy is within ± 10% when the influence of the ice is considered. Furthermore, the acceleration and displacement of the model under the separate influences of the Tianjin and Kobe seismic waves were calculated and the comparisons with test data are shown in Figs 14 and 15.

As shown in Figs 14 and 15, the calculated acceleration and displacement at the model top under the Tianjin earthquake and Kobe earthquake match the test results; however, the calculated acceleration and displacement are greater than those measured. The reason for this is that the model is an elasto-plastic body, and our proposed simplified calculation model is simplified to some extent.

**Fig 16:**
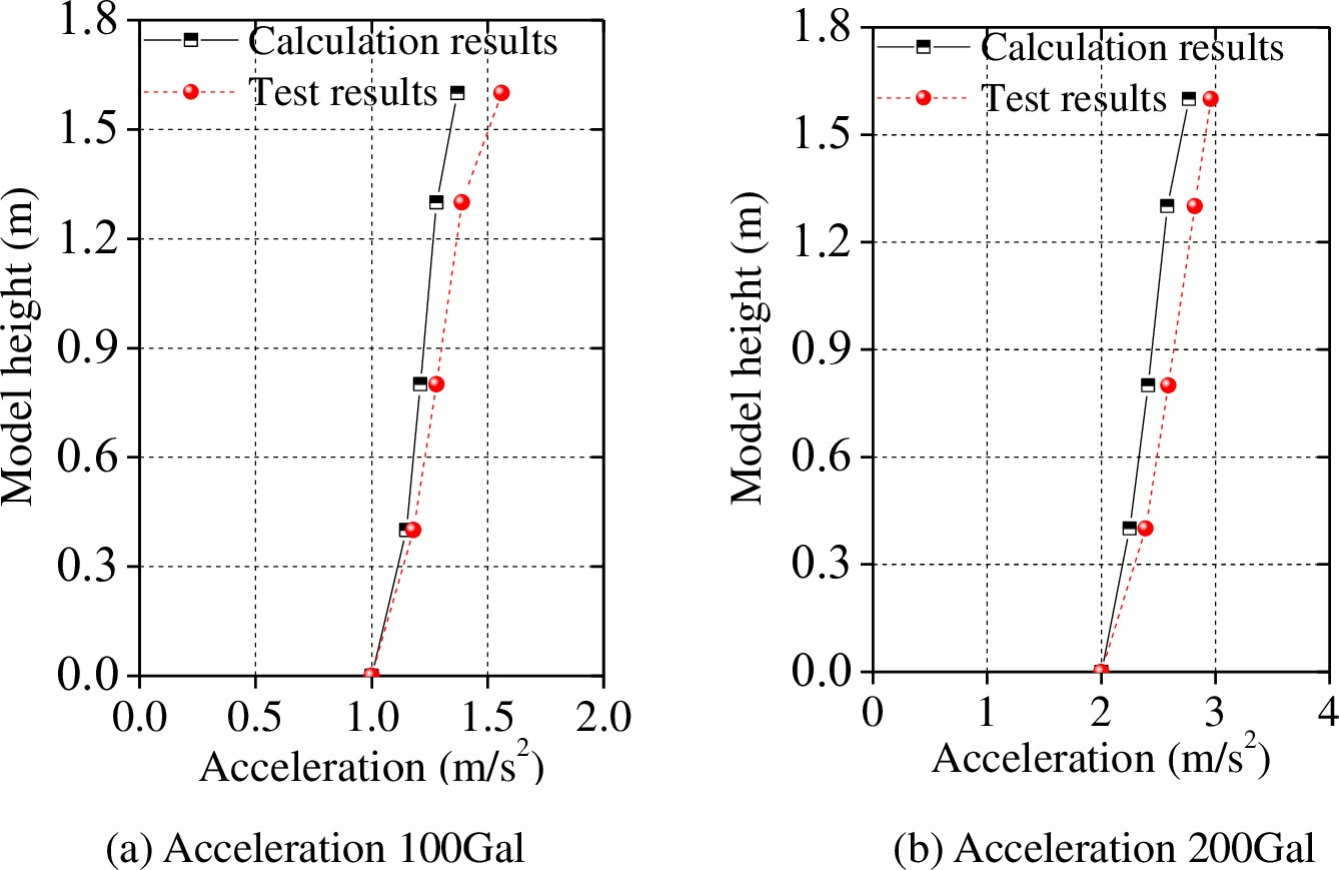
Peak acceleration along the model height: The Tianjin earthquake.

**Fig 17:**
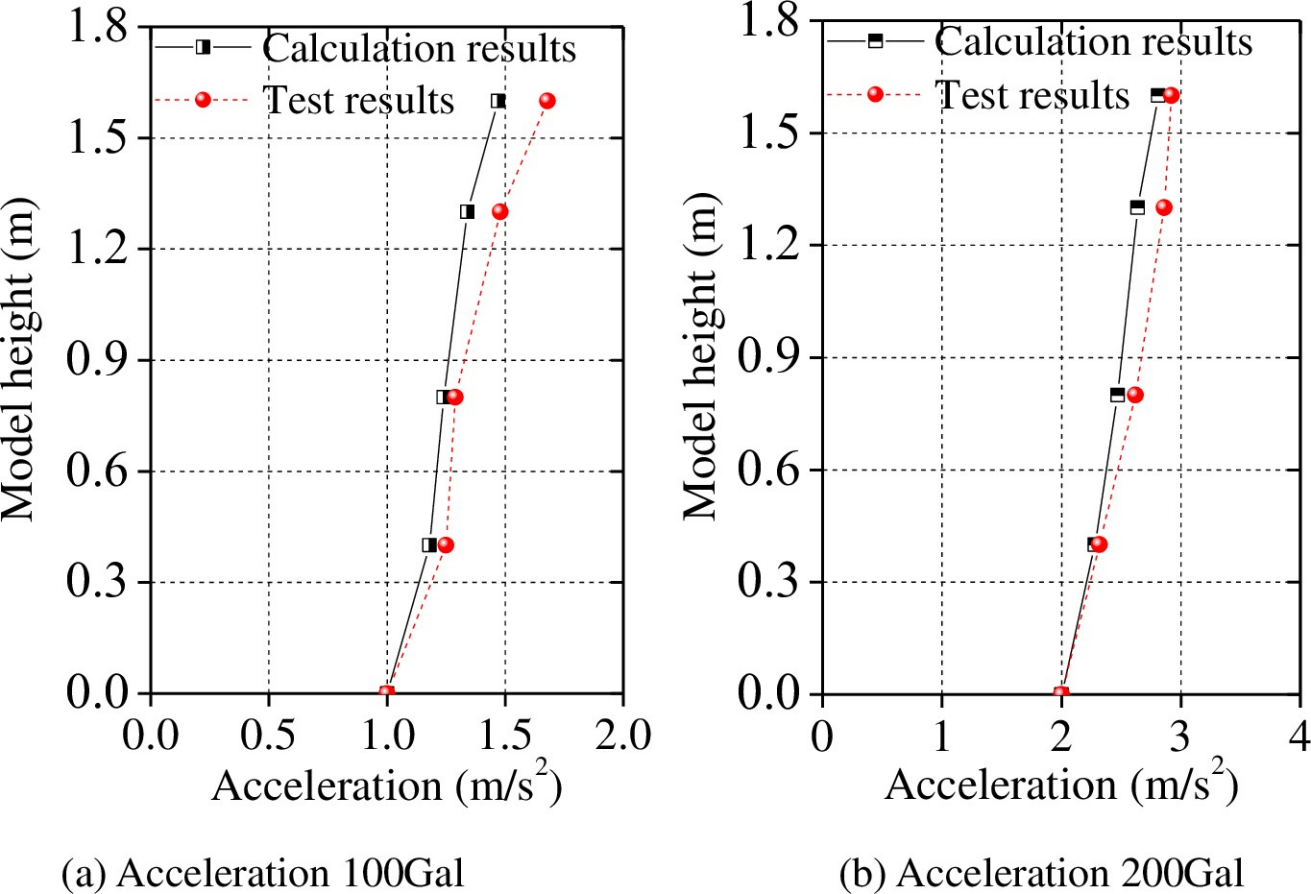
Peak acceleration along the model height: The Kobe earthquake.

Finally, we compared the calculated peak acceleration and peak displacement along the model height, and comparisons with test results are shown in Figs 16 and 17.

As shown in Figs 16 and 17, the test results of the model under seismic load are consistent with those calculated with limited differences therein under certain conditions. The maximum difference is 15.86% under Tianjin earthquake input excitation, and the maximum difference is 18.28% under Kobe earthquake conditions. In general, the trend in the distribution of the peak acceleration calculated using the proposed simplified calculation model along the height of the model is consistent with test data, suggesting that the proposed simplified calculation model is accurate.

## 4 Numerical example

### 4.1 Establishment of the calculation model

In the present study we take a monopile wind turbine tower in ice and water as the research object. The wind turbine tower is location in an active seismic zone along the east side of the south yellow sea shelf and it is close to the coast of Jiangsu province in China. The mechanical parameters of the wind turbine tower are designed according to specifications, and site soil parameters and sea ice parameters are obtained according to field tests, so the calculation results can be generalized to other turbines.

**Fig 18:**
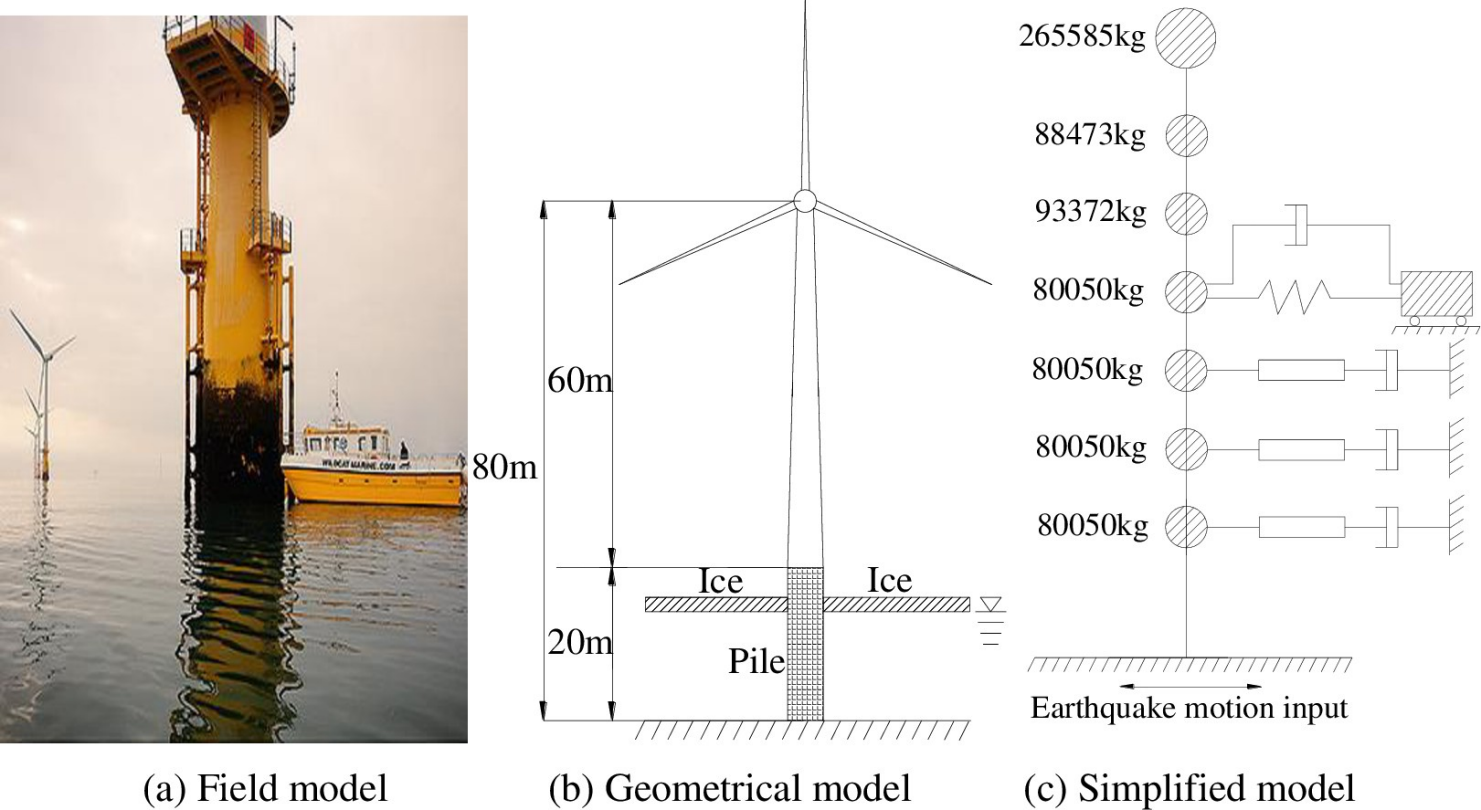
Monopile wind turbine tower diagram.

The sea water depth is 15 m, the tower height is 60 m, and the tower height above bedrock is 20 m. According to the investigation report, the ice is at this thickness 0.6m for a long time in the year. The monopile wind turbine tower is located in a Class-II site, and the seismic fortification intensity is 8 degrees. The tower body design adopts a variable-section and variable-wall-thickness cylindrical structure; the material of the tower and the tower above the ground is Q345C steel. The yield strength *σ*_y_ of the material is 345 MPa, its modulus of elasticity *E* is 2.06×10^11^ Pa and Poisson’s ratio *γ* is 0.3. The tower outer diameter is 3.07 m to 4.5 m, and the wall thickness is 18 mm to 50 mm. The compressive strength of sea ice was taken as 2.12 MPa. The mass of the ice is 900kg, the damping of the ice is 0.04, and the stiffness of the spring is 2.2Gpa. To adopt the proposed simplified calculation model, the masses of the structure were determined as shown in Fig 18.

**Fig 19:**
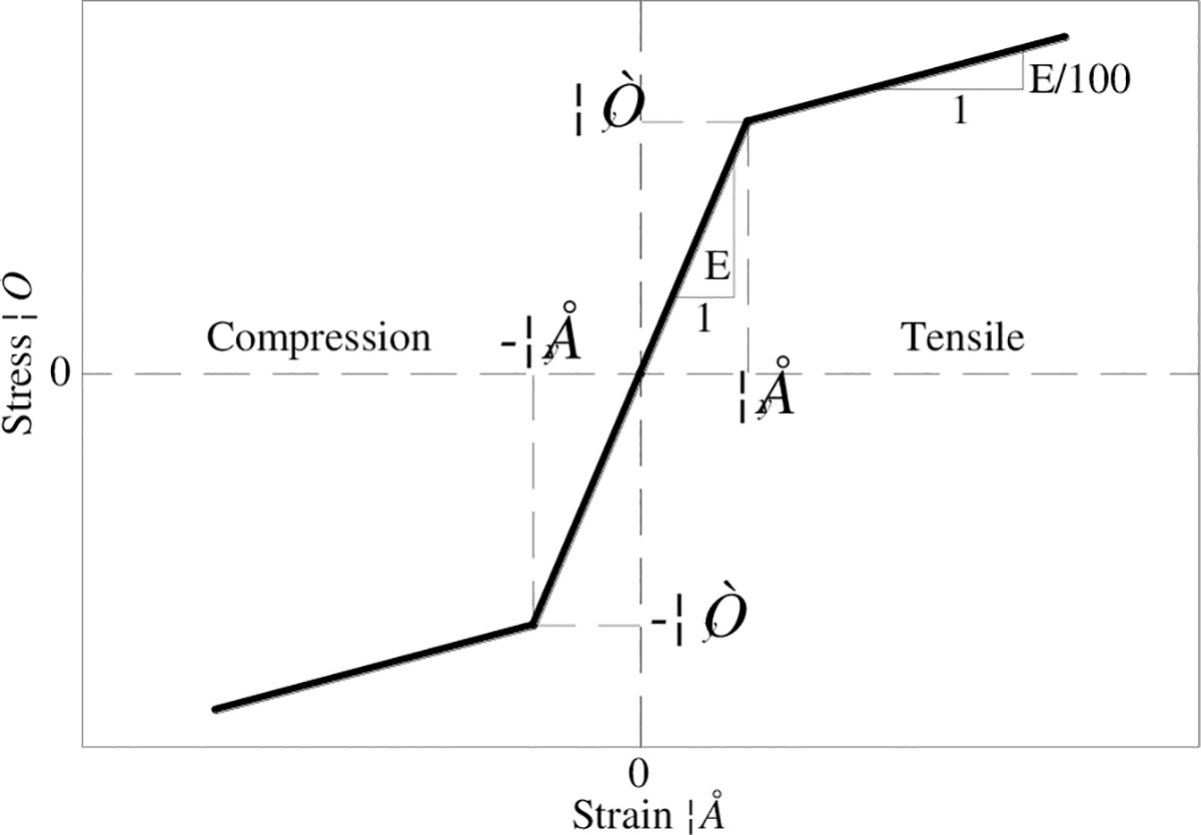
Constitutive model of the wind turbine tower.

The stress-strain relation of the steel adopts the bilinear model recommended in Japanese *Standard Specification for Highway Bridges* [5], as shown in Fig 19. As indicated in the specification, the structure is allowed some structural components to respond plastically under the rare earthquakes, but the structure is not allowed to collapse. At this time, the wind turbine will stop automatically, and this is the concept of seismic design. Therefore, this curve simplifies the plastic stage and enhanced stage of the material into a diagonal line. The corresponding elastic modulus is *E*/100.

### 4.2 Seismic waves used in this study

The basic intensity of the region of the wind turbine is 8 degrees, and the site is II. According to the *Code for Seismic Design of Buildings* (GB 50011–2010) [24] of China, the key engineering structure in accordance with the seismic fortification intensity 8 degrees needs to be considered as being at least one earthquake degree of fortification higher. In our study, the seismic fortification intensity was therefore taken to be 9 degrees, and the horizontal design peak acceleration was 400 Gal. The basic design acceleration value is shown in Table 9.

**Table 9:** Basic earthquake acceleration value in Chinese code.

Earthquake category	6 degrees	7 degrees	8 degrees	9 degrees
Design basic acceleration	0.05g	0.10g	0.20g	0.40g

**Fig 20:**
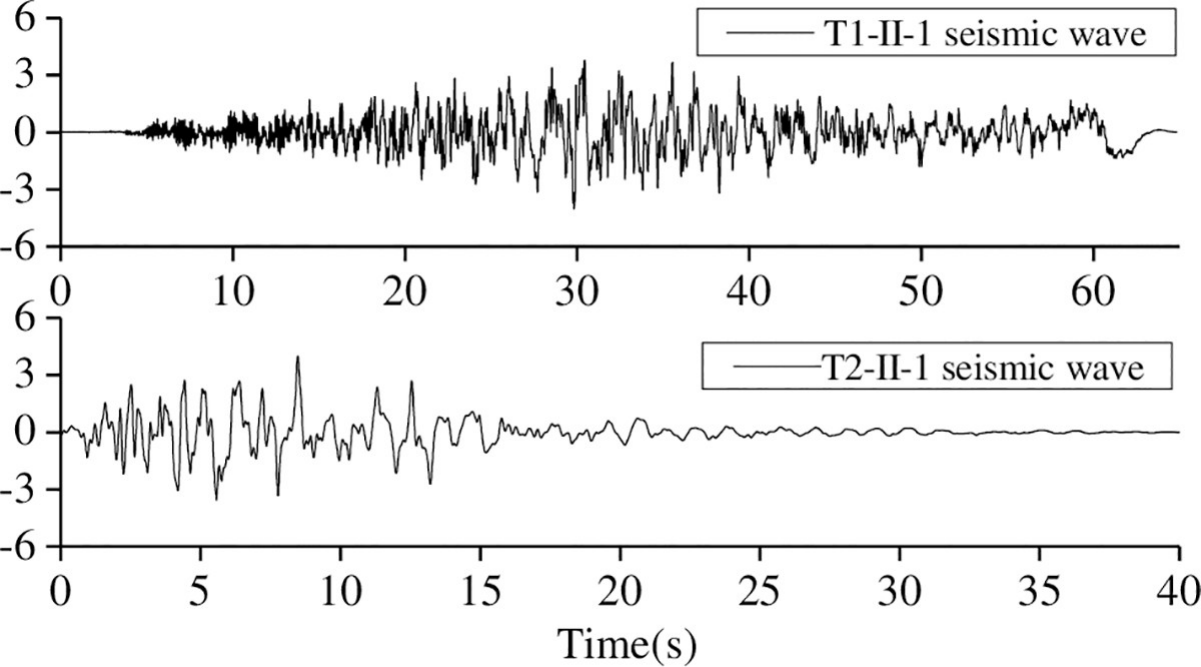
Seismic waves.

According to the Japanese code [5], near-field and far-field seismic waves (Site Type-II) were selected, and the far-field seismic waves were referred to as T1, and the near-field seismic waves were referred to as T2. The seismic waves and the basic characteristics of the seismic records are shown in Table 10 and Fig 20.

**Table 10:** Basic characteristics of the seismic records.

Type	Earthquake name	Magnitude	Epicentral Distance (km)	Record location	Epicentral distance(km)
T1-II-1	Hotan earthquake in 1968	7.5	100	Plate island bridge foundation	362.62
T2-II-1	Kobe earthquake in1995	7.2	11	TAKATORI station	686.83

### 4.3 The effects of soil type on the wind turbine tower

The soil deposits are obtained through the borehole in the site which was 40-meter depth and consisted of nine layers. Parameters of soil in this study are obtained by in-situ and laboratory tests, i.e., dynamic triaxial test and the parameters are presented in Table 11.

**Table 11:** Soil layers and design parameters.

Layer	Type	Soil thickness (m)	Physical indicators for natural state			Internal friction angle (°)	Cohesion(kPa)	Compression modulus (MPa)
			Dry density(g/cm^3^)	Unit weight	Void ratio			
1	Silty sand	6.5	1.59	2.73	0.72	32.0	3.8	11.92
2	Silty soil	1.0	1.61	2.70	0.68	30.7	9.5	11.42
3	Silty soil	3.0	1.53	2.70	0.77	30.6	8.3	11.26
4	Silty soil	5.5	1.46	2.71	0.86	27.3	11.8	7.43
5	Silty soil	4.0	1.53	2.70	0.77	30.6	8.3	11.26
6	Silty sand	5.5	1.54	2.69	0.75	32.6	3.7	11.85
7	Silty clay	3.0	1.59	2.73	0.72	18.1	41.0	5.07
8	Silty soil with silty clay	7.5	1.51	2.71	0.80	27.0	9.0	7.70
9	Silty sand	4.0	1.68	2.69	0.62	32.0	5.0	13.08

**Fig 21:**
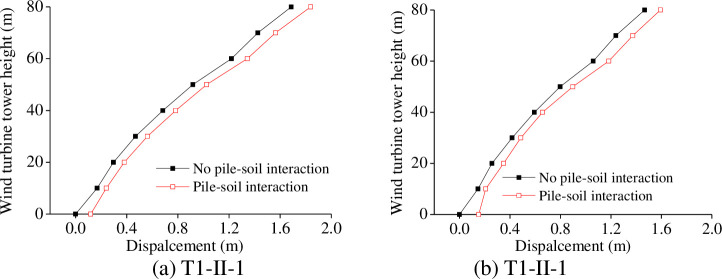
Effect of pile-soil interaction on wind turbine displacement.

In our study, *p-y* curve method is used to simulate the pile-soil interaction. The horizontal displacement is one of the main indicators in wind turbine tower design [25]; therefore, we compare the calculation results with and without the pile-soil interaction influence, as shown in Fig 21.

Fig 21 shows that there is little difference between the calculated results when the pile-soil interaction is considered or not, and the maximum error is within 12%. The change laws of displacements are consistent with the increase of wind turbine tower height. Consequently, the pile-soil interaction has little effect on the calculation results in this study.

### 4.4 Seismic performance analysis of a wind turbine tower

**Fig 22:**
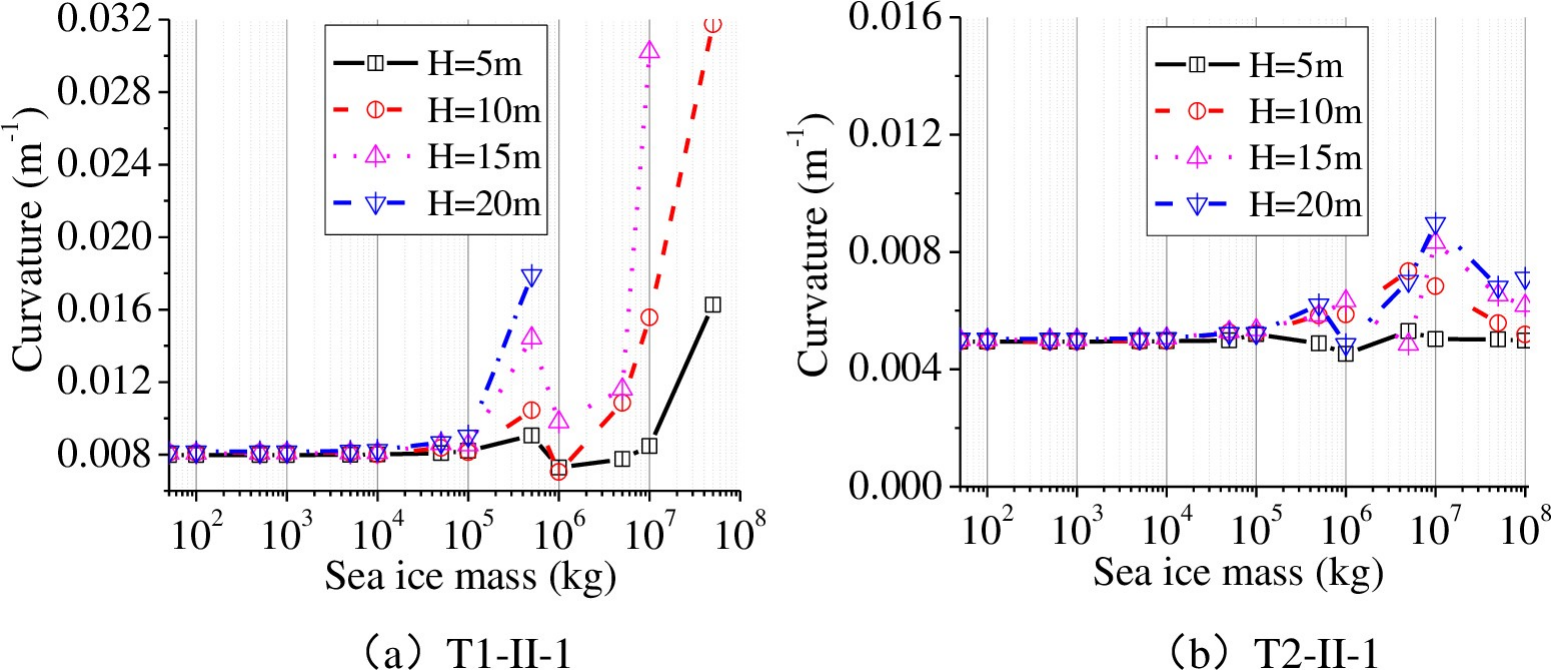
Maximum curvature of the tower bottom in different water depths.

In aseismic design of wind turbine tower, the curvature of the tower was conveniently used in analysis of damage to the tower [26], so the maximum curvature at the base of the tower to the wind turbine tower was used to study the non-linear seismic response. According to the investigation report, the ice is at this thickness 0.6m for a long time in the year. Therefore, we choose ice thickness 0.6m for seismic analysis. We set the ice thickness to 0.6 m, and analysed water depths of 5 m, 10 m, 15 m, and 20 m. The sea ice was simplified into a lumped mass in our proposed simplified model, and the changes of the maximum curvature with increasing sea ice mass are shown in Fig 22.

As shown in Fig 22, the maximum curvature at the wind turbine tower bottom under T1-II-1 earthquake action is greater than that under T2-II-1 earthquake action. Under T2-II-1 earthquake action, when the ice mass is varied from 0 kg to 1×10^8^ kg, the maximum curvature of the tower bottom under different water depths will exceed the ultimate curvature of the tower bottom section with the increase in the mass of sea ice, which means that the wind turbine is damaged. The sea ice mass, when the wind turbine tower is destroyed, decreases with increasing depth of water. For example, the curvature at the bottom of the wind turbine tower reaches a maximum when the sea ice mass is 5×10^7^ kg in water depths of 5 m and 10 m, and the curvature at the bottom of the tower reaches a maximum when the sea ice mass is 1×10^7^ kg in a water depth of 15 m, and the curvature at the bottom of the tower reaches the maximum when the sea ice mass is 5×10^6^ kg in water with a depth of 20 m. Under T2-II-1 earthquake action, the maximum curvature of the tower bottom changed significantly as the mass of sea ice increased. The least favourable curvature at the tower bottom appears when the sea ice mass ranges from 1×10^7^ kg to 5×10^7^ kg. The sea ice mass corresponding to the least favourable curvature of the tower bottom increased with the depth of the water. For example, the curvature at the bottom of the tower reaches a maximum when the sea ice mass is 5×10^7^ kg in water with a depth of 5 m, and the curvature at the bottom of the tower reaches a maximum when the sea ice mass is 1×10^7^ kg in water depths of 10 m, 15 m, and 20 m. Consequently, the sea ice mass exerts a significant influence on the maximum curvature of the bottom of the tower, and the least favourable curvature of the tower bottom mainly appears in the range from 5×10^6^ kg to 5×10^7^ kg under different types of seismic wave action, implying that a 100-m ice-plate becomes consolidated around the tower of the wind turbine tower. The effect of sea ice on the maximum curvature of the tower bottom of wind turbine tower is different in different depths of water. The sea ice mass corresponding to the maximum curvature of the tower bottom decreased with increasing depth of water under near-field earthquake (T1-II-1) excitation.

**Fig 23:**
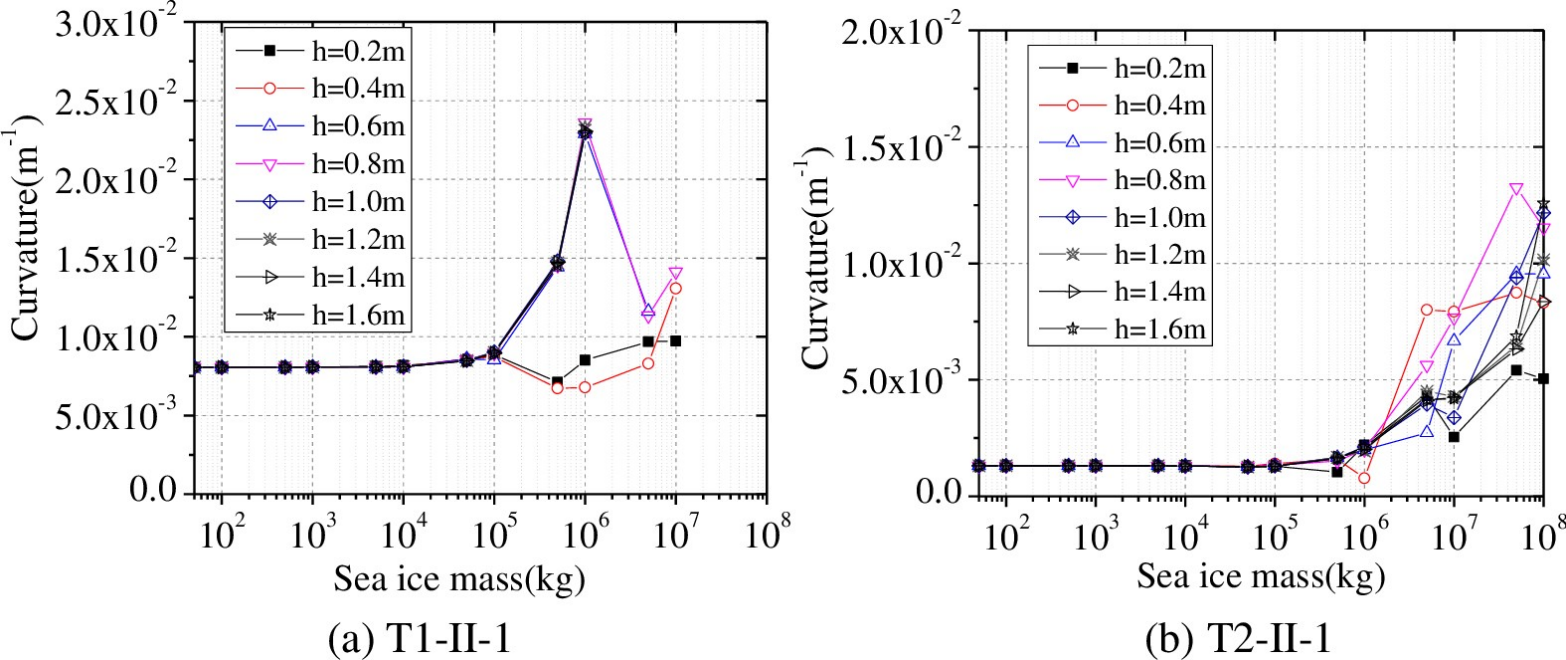
The maximum curvature of the tower bottom under earthquake action.

The thickness of the sea ice around the wind turbine tower changes with both season and temperature, so the ice thicknesses h are set to 0.2 m, 0.4 m, 0.6 m, 0.8 m, 1.0 m, 1.2 m, 1.4 m, and 1.6 m, and the water depth is set to 15 m. Changes of the maximum curvature at the tower bottom, as influenced by different ice thicknesses, are shown in Fig 23.

As shown in Fig 23, the maximum curvature influenced by different ice thicknesses is not significantly different when the ice mass is less than 1×10^6^ kg, and the maximum curvature began to change significantly with the further increase of the mass of sea ice under different seismic loads. Under T1-II-1 earthquake action, the maximum curvature is much larger than the ultimate curvature when the ice mass ranges from 0 kg to 1×10^8^ kg. Under T2-II-1 earthquake action, the maximum curvature increases with the increase of ice thickness in addition to the ice thickness of 0.8 m. Therefore, the influence of the greater mass of the sea ice on the seismic response of a wind turbine tower should be considered when the wind turbine tower is designed in an area prone to development of thick ice.

**Fig 24:**
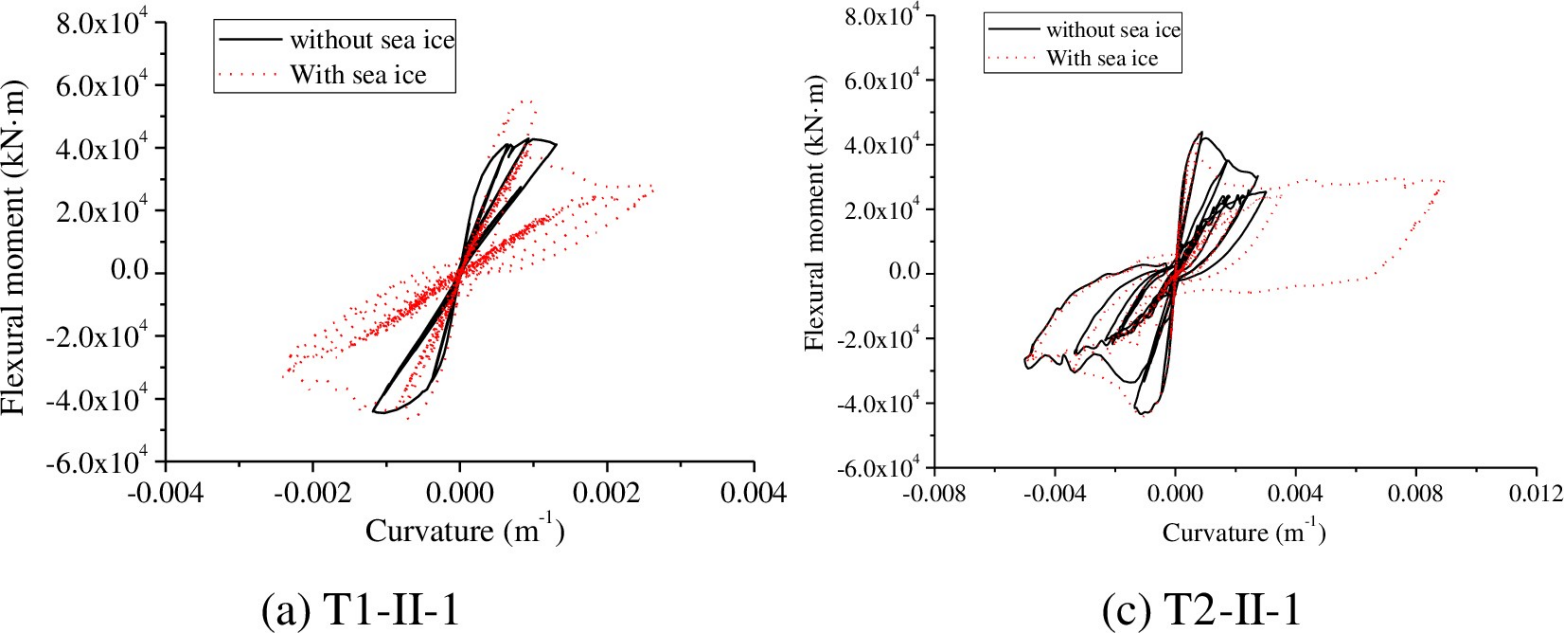
Hysteresis curves of the cross-section of the tower bottom.

From the above analysis, we found that the least favourable mass of sea ice is 1×10^7^ kg, and we set the sea mass and water depth to 1×10^7^ kg and 20 m, respectively. Then, the flexural moment and curvature hysteresis curves of the tower bottom were investigated (Fig 24).

As shown in Fig 24, the bending moment-curvature of the tower bottom with the influence is greater than that without sea ice. The bending moment-curvature hysteresis curve shows an inverted S shape more obvious than that without ice, suggesting that the deformation and energy dissipation capacity of the wind turbine tower are decreased. Therefore, the wall thickness or stiffening rib thickness should be increased to improve the seismic performance and ductility of the wind turbine tower.

**Fig 25:**
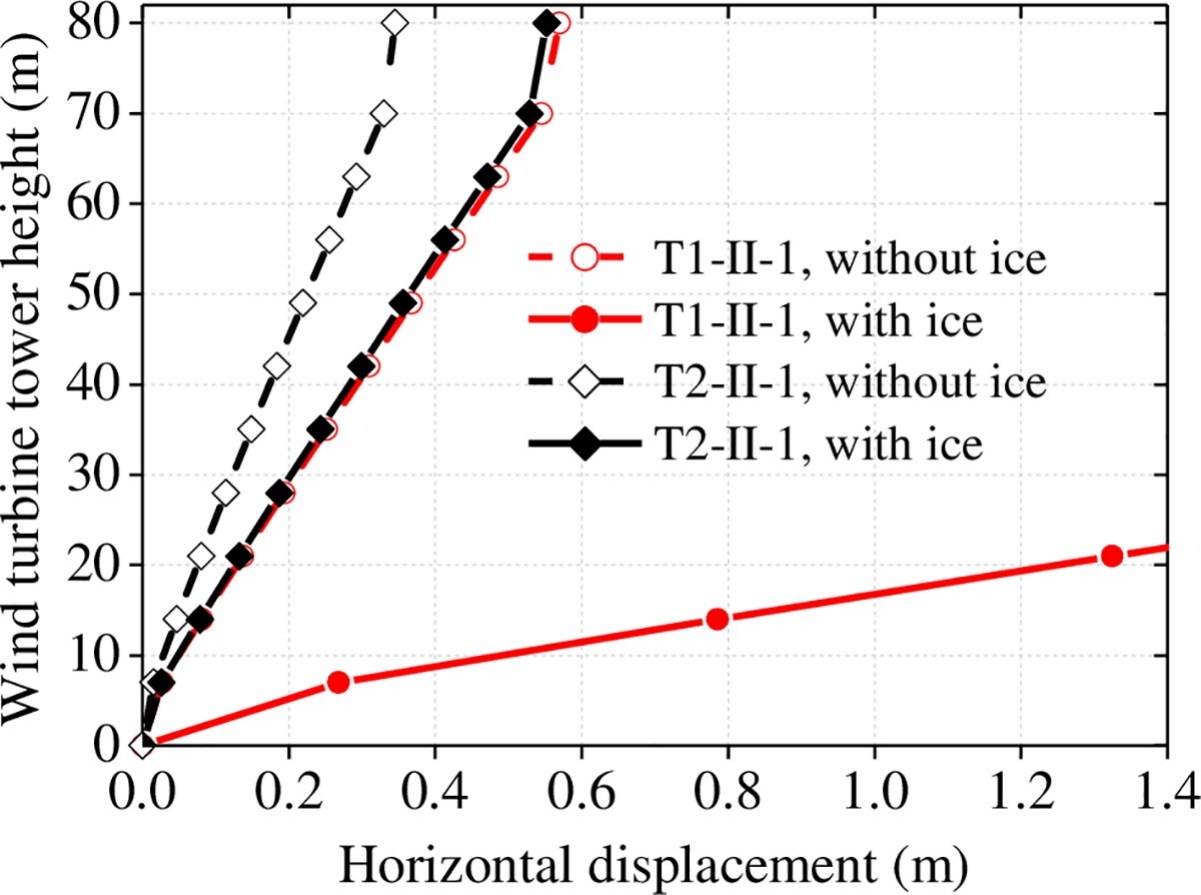
Horizontal displacement of the wind turbine tower with and without ice.

Seismic responses of wind turbine tower surrounded by sea ice can increase several times compared to those in the absence of sea ice, especially the seismic response of a wind turbine tower at point of connection. We investigated the maximum horizontal displacement, bending moment, and shearing force along the wind turbine tower height with and without influence of the sea ice, as shown in Fig 25.

**Fig 26:**
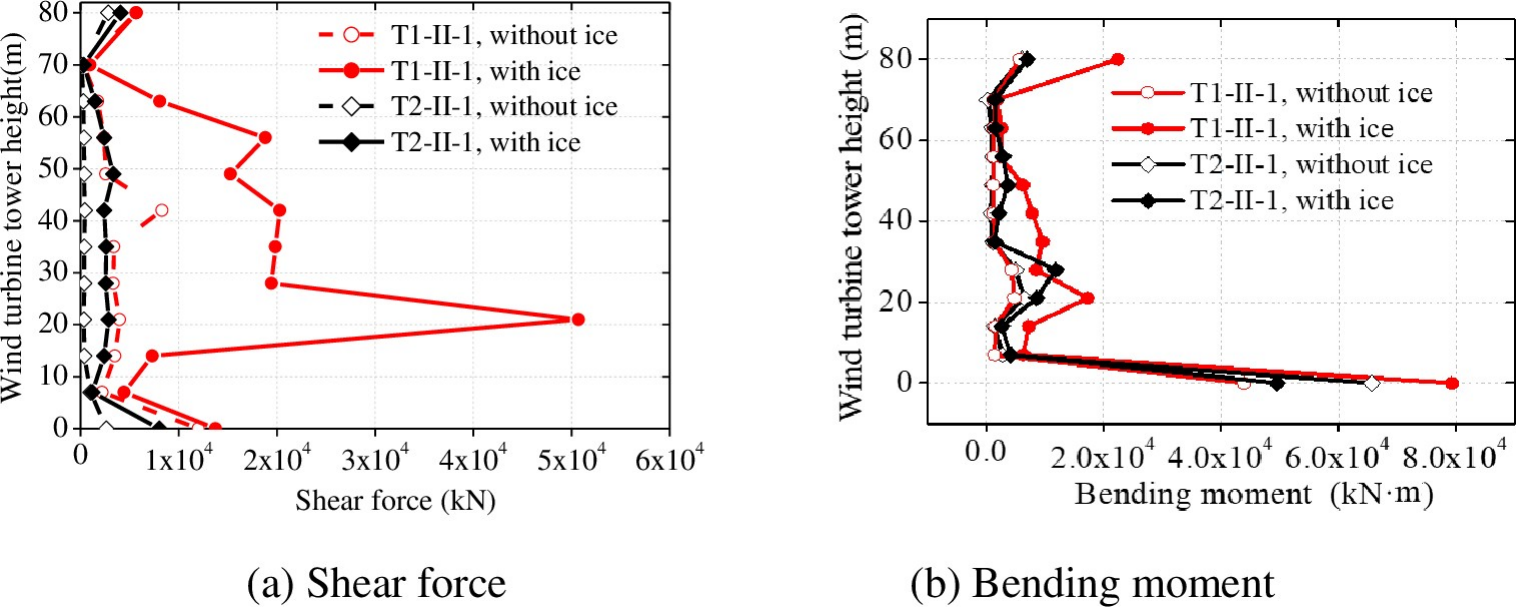
Internal force on the wind turbine tower with and without ice.

As shown in Fig 25, the wind turbine tower displacement increases significantly when affected by sea ice, and the rate of change of displacement is generally above 60% of that when affected by sea ice. In particular, the displacement of the wind turbine tower top increases by 249.5% under T1-II-1 earthquakes when affected by the sea ice. In addition, the effect of the far-field earthquake (T1-II-1) on displacement is greater than that of a near-field earthquake (T2-II-1). The internal force on the wind turbine tower with and without ice is shown in Fig 26.

Fig 26 shows that, in the presence of ice, the maximum bending moment and the maximum shear force of the wind turbine tower increase under seismic load. In particular, the maximum shear force increases at the point of interaction between sea ice and the wind turbine tower. The shear forces at the bottom of the wind turbine tower and point of action of the sea ice increased by 221.7% and 374.4% respectively. The maximum bending moment also increased significantly at the bottom of the wind turbine tower and point of action of the sea ice: because the sea ice, as a part of the wind turbine tower system, moves with the structure and increases the horizontal seismic force; thus, the wind turbine tower is subjected to a horizontal shear force. Compared to conditions without ice, the shear force on the wind turbine tower changes at the bottom of the wind turbine tower and point of action of the sea ice, and attention should be paid to this in wind turbine tower design. Therefore, in the seismic design of a wind turbine tower, it is necessary to carry out anti-seismic check calculations on the wind turbine tower bottom and the point of action of the sea ice when the wind turbine tower is affected by the least favourable sea ice mass; it is also necessary to add a stiffener in this position to ensure the safety of the wind turbine tower when it becomes surrounded by sea ice and subject to an earthquake.

## 5 Conclusion

A simplified model of the water-ice-structure dynamic interaction was proposed to investigate the effect of the sea ice on the seismic behaviour of a wind turbine tower, and the novelties of the study are as follows:

1. A simplified seismic response analysis model of a wind turbine tower, ice, and water under earthquake load was established, and this obviated the need to solve complicated non-linear equations and reduced the computational burden to a significant extent.
2. The energy dissipation capacity of the wind turbine tower was decreased by the effects of the sea ice, and the mass of sea ice exerted a significant influence on the curvature at the wind turbine power bottom, which could reflect the plastic deformation of the wind turbine tower. The curvature began to change significantly under different earthquake loads when the ice mass exceeded 1×10^6^ kg; therefore, the wall thickness or stiffening rib thickness should be increased to improve the seismic performance and ductility of the wind turbine tower.
3. The maximum bending moment and shear force increased significantly at the bottom of the wind turbine tower and the action point of the sea ice, thus, in the seismic design of such wind turbine towers, it was deemed necessary to conduct anti-seismic design check-calculations on the wind turbine tower bottom and the action point of the sea ice. According to the design principle of the similarity ratio, paraffin ice was used to simulate sea ice and shaking table tests were conducted to verify the accuracy of the calculated results using our proposed simplified model: the calculated results were within accepted tolerances.
